# Random generation of RNA secondary structures according to native distributions

**DOI:** 10.1186/1748-7188-6-24

**Published:** 2011-10-12

**Authors:** Markus E Nebel, Anika Scheid, Frank Weinberg

**Affiliations:** 1 Department of Computer Science, University of Kaiserslautern, Germany

**Keywords:** Random generation, stochastic context-free grammars, RNA secondary structures

## Abstract

**Background:**

Random biological sequences are a topic of great interest in genome analysis since, according to a powerful paradigm, they represent the *background noise *from which the actual biological information must differentiate. Accordingly, the generation of random sequences has been investigated for a long time. Similarly, random object of a more complicated structure like RNA molecules or proteins are of interest.

**Results:**

In this article, we present a new general framework for deriving algorithms for the non-uniform random generation of combinatorial objects according to the encoding and probability distribution implied by a stochastic context-free grammar. Briefly, the framework extends on the well-known recursive method for (uniform) random generation and uses the popular framework of admissible specifications of combinatorial classes, introducing weighted combinatorial classes to allow for the non-uniform generation by means of unranking. This framework is used to derive an algorithm for the generation of RNA secondary structures of a given fixed size. We address the random generation of these structures according to a realistic distribution obtained from real-life data by using a very detailed context-free grammar (that models the class of RNA secondary structures by distinguishing between all known motifs in RNA structure). Compared to well-known sampling approaches used in several structure prediction tools (such as SFold) ours has two major advantages: Firstly, after a preprocessing step in time $O\left(n^{2}\right)$ for the computation of all weighted class sizes needed, with our approach a set of *m *random secondary structures of a given structure size *n *can be computed in worst-case time complexity $O\left(m\cdot n\cdot \log\left(n\right)\right)$ while other algorithms typically have a runtime in $O\left(m\cdot n^{2}\right)$. Secondly, our approach works with integer arithmetic only which is faster and saves us from all the discomforting details of using floating point arithmetic with logarithmized probabilities.

**Conclusion:**

A number of experimental results shows that our random generation method produces realistic output, at least with respect to the appearance of the different structural motifs. The algorithm is available as a webservice at http://wwwagak.cs.uni-kl.de/NonUniRandGen and can be used for generating random secondary structures of any specified RNA type. A link to download an implementation of our method (in Wolfram Mathematica) can be found there, too.

## Background and Introduction

The topic of random generation algorithms (also called *samplers*) has been widely studied by computer scientists. As stated in [1], it has been examined under different perspectives, including combinatorics, algorithmics (design and/or engineering), as well as probability theory, where two of the main motivations for random sampling are the testing of combinatorial properties of structures (e.g. conjectured structural properties, quantitative aspects), as well as the testing of properties of the corresponding algorithms (with respect to correctness and/or efficiency).

As considers software engineering, the so-called *random testing *approach is commonly used to test implementations of particular algorithms, as it is usually not feasible to consider all possible inputs and unknown which of these inputs are among the most interesting ones. In fact, this approach requires for the generation of random instances of program inputs that obey various sorts of syntactic and semantic constraints (where the random instances usually ought to be of a preliminarily fixed input size in order to be comparable to each other).

**Figure 1:**
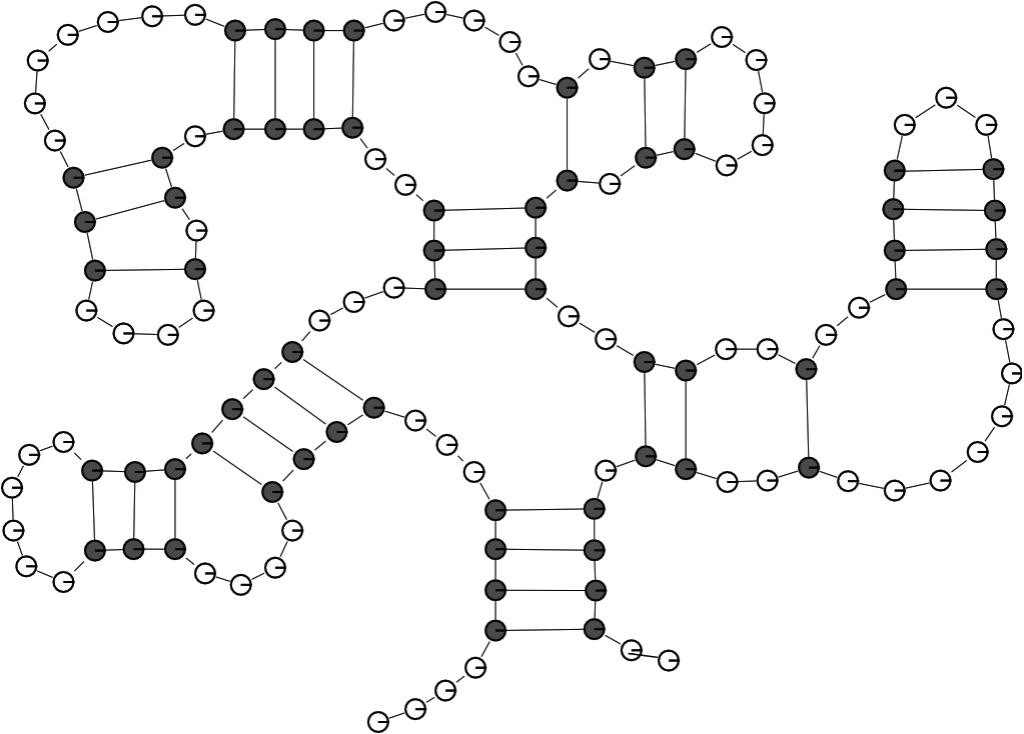
**An RNA secondary structure**. Unpaired and paired bases are represented by white and gray points, respectively.

In the Bioinformatics area, algorithms for generating random biological sequences have been investigated for a long time (see e.g. [2,3]). As stated in [4], random sequences are a topic of great interest in genome analysis, since according to a powerful paradigm, they represent the *background noise *from which the actual biological information must differentiate. Thus, random generation of combinatorial objects can be used in this context for simulations studies in order to isolate signal (unexpected events) from noise (statistically unavoidable regularities). In fact, according to [4], random biological sequences are for instance widely used for the detection of over-represented and under-represented motifs, as well as for determining whether scores of pairwise alignments are relevant or not: although there exist analytic approaches for these kinds of problems, for the most complex cases, it is often still necessary to be able to alternatively use a corresponding experimental approach (based on randomly generated sequences obtained from a computer programm). For this purpose, random sequences must obviously obey to a certain model that takes into account some relevant properties of actual real-life sequences, where such models are usually based on statistical parameters only. However, it is known that these classical models can be enriched by adding structural parameters (see [4]). Over the past years, several methods have been proposed for the random generation of more complex structures, where special attention has been paid to *RNA secondary structures*. RNA is a single-stranded nucleotide polymer and a major component of cellular processes (like DNA and proteins). An RNA strand is formed by linking together certain nucleotide units. The specific sequence of nucleotides along this chain is called the *primary structure *of the molecule. By pairing of nucleotides that are not linked in this chain (i.e. by the so-called effects of base pairing), the linear primary structure is folded into a three-dimensional conformation, called the *tertiary structure*, which in many cases determines the function of the molecule. Most of the 3D structure is determined by the intramolecular base-pairing interactions *in the plane*, which together form the *secondary structure *of the molecule. For this reason, *pseudoknots *(induced by crossing base pairs) are considered as tertiary interactions and are usually not permitted in the definition of secondary structure. As unknotted structures contain only nested base pairs and are thus essentially two-dimensional, they can be modeled as planar graphs. This rather descriptive and commonly used planar graph model for RNA secondary structures was first formalized in [5]. An example is shown in Figure 1.

Most of the existing random generation algorithms for RNA secondary structures are used for predicting the structure of a given RNA sequence (see e.g. [6,7]), while others can be employed for instance for evaluating structure comparison softwares [8]. Note that secondary structure prediction methods based on random sampling represent a non-deterministic counterpart to the up-to-date most successful and popular physics-based prediction methods that make use of the energy minimization paradigm and are realized by dynamic programming algorithms (see e.g. [9-12]). Random sampling also differs from the stochastical RNA structure prediction approach that is based on context-free modeling of structural motifs and adding some statistical parameters observed in real-life data by assigning probabilities to the corresponding motifs (see e.g. [13-15]). Nevertheless, it should be mentioned that statistical sampling methods like [6,7] used for RNA structure prediction are based on thermodynamics and thus inevitably inherit the problems and imprecisions related to energy minimizing methods, which are caused by the still incomplete commonly used free energy models for RNAs. In order to overcome these pitfalls, one could take the competing point of view and consider only typical structural information observed in a set of sample data as the basis for a new random generation method. If that information draws a realistic picture for all the different motifs of a molecule's folding, the corresponding sampling method is likely to produce realistic results. Accordingly, several authors made use of stochastic context free grammars and employed machine-learning techniques to train parameter values from a set of known secondary structures. Such grammars have widely been used in a predictive mode (see, e.g., [14]) but there are also successful examples of applications where the random sampling of derivation trees has been the core of the method (see, e.g., [16-18] but also [19] for examples). In the present paper, we follow that line of ideas and rely on the approach of the technical report [20] to develop a new algorithm for the (non-uniform) random generation of RNA secondary structures (without pseudoknots) according to a distribution induced by a set of sample RNA data (note that the algorithm actually generates secondary structures for a preliminary fixed size, *not *for a given RNA sequence of this size, which means we take the combinatorial point of view and completely abstract from sequence).

The main contribution of this manuscript is the derivation of a new and efficient algorithm for the random generation of RNA secondary structures according to an elaborate and thus very realistic model. For this purpose we use and generalize the approach from [20]. Particularly, our random generation method is based on a sophisticated context-free grammar for unknotted structures which, in order to model the class of all considered RNA secondary structures as realistic as possible, distinguishes between all known structural motifs that may occur in unknotted RNA secondary structure. This means that any structural feature is modeled by one or more specific grammar rules with corresponding probabilities observed from real-life data. Note that this grammar is actually a special variant of the comprehensive grammar used in [21] for deriving a realistic RNA structure model and for performing the first ever analytical analysis of the expected free energy of a random secondary structure (of a specified RNA type). Actually, that grammar has been designed as a mirror of the famous Turner energy model [22,23] which serves as the foundation for most of the existing physics-based RNA structure prediction methods: all structural motifs for which there are different thermodynamic rules and parameters are created by distinct production rules (with corresponding probabilities).

According to [20], our sampling method involves a weighted *unranking *algorithm for obtaining the final structures. Briefly, considering an arbitrary structure class of size (cardinality) *c*, a corresponding unranking method uses a well-defined ordering of all class elements (according to a particular numbering scheme, the so-called *ranking *method) and for a given input number *r *∈ {1,..., *c*} outputs the structure with rank *r *in the considered ordering. That way, the random sampling based on a stochastic grammar - building heavily on the use of small floating point numbers - is translated into an unranking algorithm using integer values only. Notably, a complete structure of size *n *is generated by recursively unranking the distinct structural components from the corresponding subclasses (of substructures with sizes less than *n*). In our case, the weighted unranking algorithm requires a precomputation step in worst-case time $O\left(n^{2}\right)$ for computing all weighted class sizes up to input size *n*. The worst-case complexity for generating a secondary structure of size *n *at random is then given by O(nlogn) since we are ranking structures according to the *boustrophedon order *(see e.g. [7]).

By the end of this paper, we analyze the quality of randomly generated structures by considering some experimental results. First, we will consider statistical indicators of many important parameters related to particular structural motifs and compare the ones observed in the used sample set of real world RNA data to those observed in a corresponding set of random structures. Their comparison measures indicate that our method actually generates realistic RNA structures. Obviously, an algorithm which, for a given structure size n, produces random RNA secondary structures that are - related to expected shapes of such structures -in most cases realistic is a major improvement over existing approaches which, for example, are only capable of generating secondary structures uniformly for size *n*. Furthermore, we will consider the two different free energy models defined in [21] for RNA secondary structure (with unknown RNA sequence) to get further evidence of the good quality of our random generation method (with respect to free energies and thus rather likely also with respect to appearance of the different structural motifs of RNA).

## Prior Results and Basic Definitions

### Uniform Random Generation

In the past, the problem of *uniform *random generation of combinatorial structures, that is the problem of randomly generating objects (of a preliminary fixed input size) of a specified class that have the same or similar properties, has been extensively studied. Special attention has been paid on the wide class of *decomposable structures *which are basically defined as combinatorial structures that can be constructed recursively in an unambiguous way.

In principle, two general (systematic) approaches have been developed for the uniform generation of these structures: First, the *recursive method *originated in [24] (to generate various data structures) and later systematized and extended in [25] (to decomposable data structures), where general combinatorial decompositions are used to generate objects at random based on counting possibilities. Second and more recently, the so-called *Boltzmann method *[1,26], where random objects (under the corresponding Boltzmann model) have a fluctuating size, but objects with the same size invariably occur with the same probability. Note that according to [26], Boltzmann samplers may be employed for approximate-size (objects with a randomly varying size are drawn) as well as fixed-size (objects of a strictly fixed size are drawn) random generation and are an alternative to standard combinatorial generators based on the recursive method. However, fixed-size generation is considered the standard paradigm for the random generation of combinatorial structures.

### (Admissible) Constructions and Specifications

According to [25], a decomposable structure is a structure that admits an equivalent *combinatorial specification*:

**Definition 0.1 **( [25]). Let $A=\left(A_{1},...,A_{r}\right)$ be an *r*-tuple of classes of combinatorial structures. A *specification *for  is a collection or *r *equations with the *i*th equation being of the form $A_{i}=\phi_{i}\left(A_{1},...,A_{r}\right)$, where *ϕ*_*i *_denotes a term built of the $A_{j}$ using the *constructions *of disjoint union, cartesian product, sequence, set and cycle, as well as the initial (neutral and atomic) classes.

The needed formalities that will also be used in the sequel are given as follows:

**Definition 0.2 **( [27]). If  is a combinatorial class, then $A^{n}$ denotes the class of objects in  that have size (defined as number of atoms) *n*. Furthermore:

• Objects of size 0 are called *neutral objects *or *tags *and a class consisting of a single neutral object *ϵ *is called a *neutral class*, which will be denoted by *ε *(*ε*_1_*, ε*_2_,... to distinguish multiple neutral classes containing the objects *ϵ*_1_, *ϵ*_2_, ..., respectively).

• Objects of size 1 are called *atomic objects *or *atoms *and a class consisting of a single atomic object is called an *atomic class*, which will be denoted by Ƶ(Ƶ_*a*_, Ƶ_*b*_,... to distinguish the classes containing the atoms *a*,*b*,..., respectively).

• If $A_{1},...,A_{k}$ are combinatorial classes and *ϵ*_1_, ..., *ϵ*_*k *_are neutral objects, the *combinatorial sum *or *disjoint union *is defined as $A_{1}+...+A_{k}:=\left(\varepsilon_{1}\times A_{1}\right)\cup ...\cup \left(\varepsilon_{k}\times A_{k}\right)$ where ∪ denotes set theoretic union.

• If  and  are combinatorial classes, the *cartesian product *is defined as $A\times B:=\left\{\left(\alpha ,\beta \right)|\alpha \in A\;\text{and}\;\beta \in B\right\}$, where size(*α*, *β*) = size(*α*) + size(*β*).

Note that the constructions of disjoint union, cartesian product, sequence, set and cycle are all admissible:

**Definition 0.3 **( [27]). Let  ϕ be an *m*-ary construction that associates to a any collection of classes $B_{1},...,B_{m}$ a new class $A:=\phi \left[B_{1},...,B_{m}\right]$. The construction  ϕ is *admissible *iff the counting sequence (*a*_*n*_) of  only depends on the counting sequences (*b*_1,*n*_),..., (*b*_*m*,*n*_) of $B_{1},...,B_{m}$, where the counting sequence of a combinatorial class  is the sequence of integers (*a*_*n*_)_n≥0 _for $a_{n}=\text{card}\left(A^{n}\right)$.

The framework of (admissible) specifications obviously resembles that of *context-free grammars (CFGs) *known from formal language theory (note that we assume the reader has basic knowledge of the notions concerning context-free languages and grammars. An introduction can be found for instance in [28]). In order to translate a CFG into the framework of admissible constructions, it is sufficient to make each terminal symbol an atom and to assume each non-terminal *A *to represent a class  (the set of all words which can be derived from non-terminal *A*). However, for representing CFGs, only the admissible constructions disjoint union, cartesian product and sequence are needed: Words are constructed as cartesian products of atoms, sentential forms as cartesian products of atoms and the classes assigned to the corresponding non-terminal symbols. For instance, a production rule *A *→ *aB *translates into the symbolic equation A=a×B. Different production rules with the same left-hand side give rise to the union of the corresponding cartesian products. Nevertheless, it should be noted that [25] also shows how to reduce specifications to *standard form*, where the corresponding standard specifications constitute the basis of the recursive method for uniform random generation and extends the usual *Chomsky normal form (CNF) *for CFGs. Briefly, in standard specifications, all sums and products are binary and the constructions of sequences, sets and cycles are actually replaced with other constructions (for details see [25]).

The prime advantage of standard specifications is that they translate directly into procedures for computing the sizes of all combinatorial subclasses of the considered class  of combinatorial objects. This means they can be used to count the number of structures of a given size that are generated from a given non-terminal symbol. Moreover, standard specifications immediately translate into procedures for generating one such structure uniformly at random. The corresponding procedures (for class size calculations and structure generations) are actually required for (uniform) random generation of words of a given CFG by means of unranking.

Simply speaking, the unranking of decomposable structures (like for instance RNA secondary structures which can be uniquely decomposed into distinct structural components) works as follows: Each structure *s *in the combinatorial class $S^{n}$ of all feasible structures having size *n *is given a number (rank) $i\in \left\{0,...,\text{card}\left(S^{n}\right)-1\right\}$, defined by a particular ranking method. Based on this ordering of the considered structure class $S^{n}$, the corresponding unranking algorithm for a given input number $i\in \left\{0,...,\text{card}\left(S^{n}\right)-1\right\}$ computes the single structure $s\in S^{n}$ having number *i *in the ranking scheme defined for class $S^{n}$.

Note that in this context of unranking particular elements from a considered structure class, the corresponding algorithms make heavy use of their decomposability, as the distinct structural components are unranked from the corresponding subclasses. In fact, the class sizes can be derived according to the following recursion:

$$\text{size(}C,n\text{): = }\left\{\begin{array}{cc} 1 & C\;\text{is}\;\text{neutral}\;\text{and}\;n=0,\\ 0 & C\;\text{is}\;\text{neutral}\;\text{and}\;n\neq 0,\\ 1 & C\;\text{is}\;\text{atomic}\;\text{and}\;n=1,\\ 0 & C\;\text{is}\;\text{atomic}\;\text{and}\;n\neq 1,\\ \sum_{i=1}^{k}\text{size}\left(A_{i},n\right) & C=A_{1}+...+A_{k},\\ \sum_{j=0}^{n}size\left(A,j\right)\cdot \text{size}\left(B,n-j\right) & C=A\times B. \end{array}\right)$$

Note that when computing the sums for cartesian products, we can either consider the values for *j *in the *sequential *(also called *lexicographic*) order (1, 2, 3,..., *n*) or in the so-called *boustrophedon *order $\left(1,n,2,n-1,...,\left\lceil \frac{n}{2}\right\rceil \right)$. In either case, given a fix number of considered combinatorial (sub)classes (or corresponding non-terminal symbols), the precomputation of all class size tables up to size *n *requires $O\left(n^{2}\right)$ operations on coefficients. One random generation step then needs $O\left(n^{2}\right)$ arithmetic operations when using the *sequential method *and $O\left(n\cdot \log\left(n\right)\right)$ operations when using the *boustrophedon method *(for details we refer to [25]). Obviously, using uniform unranking procedures to construct the *i*th structure of size *n *for a randomly drawn number *i*, any structure of size *n *is equiprobably generated. Consequently, in order to make sure that, for given size *n *and a sample set of random numbers *i*, the corresponding structures are in accordance with an appropriate probability distribution (as for instance observed from real-life RNA data), it is mandatory to use a corresponding non-uniform unranking method or an alternative non-uniform random generation approach.

### Non-Uniform Random Generation

Coming back to the random testing problem from software engineering, we observe that generating objects of a given class of input data according to a uniform distribution is sufficient for testing the correctness of particular algorithms. However, if one intends to gather information about the "real-life behaviour" of the algorithm (e.g. with respect to runtime or space requirements), we need to perform simulations with input data that are as closely as possible related to corresponding application. This means to obtain suitable test data, we need to specify a distribution on the considered class that is similar to the one observed in real life and draw objects at random according to this (non-uniform) distribution. Deriving such a "realistic" distribution on a given class of objects can easily be done by modeling the class by an appropriate *stochastic context-free grammar (SCFG)*. Details will follow in the next section.

As regards RNA, it has been proven that both the combinatorial model (that is based on a uniform distribution such that all structures of a given size are equiprobable and that completely abstracts from the primary structure, see e.g. [29-31]) and the Bernoulli-model (which is capable of incorporating information on the possible RNA sequences for a given secondary structure, see e.g. [32-34]) for RNA secondary structures are rather unrealistic. However, modeling these structures by an appropriate SCFG yields a more realistic RNA model, where the probability distribution on all structures is determined from a database of real world RNA data (see e.g. [35,36]).

Based on this observation, the problem of *non-uniform *random generation of combinatorial structures has been recently addressed in [20]. There, it is described how to get algorithms for the random generation of objects of a previously fixed size according to an arbitrary (non-uniform) distribution implied by a given SCFG. In principle, the construction scheme introduced in [20] extends on the recursive method for the (uniform) random generation [25] and adapted it to the problem of unranking of [37]: the basic principle is that any (complex) combinatorial class can be decomposed into (or can be constructed from) simpler classes by using admissible constructions.

Essentially, in [20], a new admissible construction called *weighting *has been introduced in order to make non-uniform random generation possible. By weighting, we understand the generation of distinguishable copies of objects. Formally:

**Definition 0.4**. If  is a combinatorial class and λ is an integer, the *weighting *of  by λ is defined as $\lambda A:=\underset{\lambda \text{ times}}{\underset{︸}{A+\ldots +A}}$. We will call two objects from a combinatorial class *copies of the same object *iff they only differ in the tags added by weighting operations.

For example, if we weight the class A={a} by two, we assume the result to be the set {*a*, *a*}; weighting B={b} by three generates {*b*,*b*,*b*}. Thus, 2A+3B={a,a,b,b,b} and within this class, *a *has relative frequency $\frac{2}{5}$, while *b *has relative frequency $\frac{3}{5}$. Hence, this way it becomes possible to regard non-uniformly distributed classes.

As weighting a class can be replaced by a disjoint union, $\text{size}\left(\lambda A,n\right)=\lambda \cdot \text{size}\left(A,n\right)$ and the complexity results from [37] also hold for weighted classes. Hence, the corresponding class size computations up to *n *need $O\left(n^{2}\right)$ time.

### Stochastic Context-Free Grammars

As already mentioned, *stochastic context-free grammars (SCFGs) *are a powerful tool for modeling combinatorial classes and the essence of the non-uniform random sampling approach that will be worked out in this article. Therefore, we will now give the needed background information.

#### Basic Concepts

Briefly, SCFGs are an extension of traditional CFGs: usual CFGs are only capable of modeling the class of all generated structures and thus inevitably induce a uniform distribution on the objects, while SCFGs additionally produce a (non-uniform) probability distribution on the considered class of objects. In fact, an SCFG is derived by equipping the productions of a corresponding CFG with probabilities such that the induced distribution on the generated language models as closely as possible the distribution of the sample data.

The needed formalities are given as follows:

**Definition 0.5 **( [38]). A *weighted context-free grammar (WCFG) *is a 5-tuple $G=\left(I,T,R,S,W\right)$, where *I *(resp. *T*) is an alphabet (finite set) of intermediate (resp. terminal) symbols (*I *and *T *are disjoint), *S *∈ *I *is a distinguished intermediate symbol called *axiom, R *⊂ *I *× (*I *∪ *T*)* is a finite set of production rules and *W *: *R *→ ℝ^+ ^is a mapping such that each rule *f *∈ *R *is equipped with a weight *w*_*f *_: = *W*(*f*). If  is a WCFG, then  is a *stochastic context-free grammar (SCFG) *iff the following additional restrictions hold:

1. For all *f *∈ *R*, we have *W*(*f*) ∈ (0,1], which means the weights are probabilities.

2. The probabilities are chosen in such a way that for all *A *∈ *I*, we have $\sum_{f\in R,Q\left(f\right)=A}w_{f}=1$, where *Q*(*f*) denotes the *premise *of the production *f*, i.e. the first component *A *of a production rule (*A*, *α*) ∈ *R*. In the sequel, we will write *w*_*f *_: *A *→ *α *instead of *f *= (*A*, *α*) ∈ *R*, *w*_*f *_= *w*(*f*).

However, at this point, we decided to not recall the basic concepts regarding SCFGs, as they are not really necessary for the understanding of this article. The interested reader is referred to the corresponding section in [21]. For a more fundamental introduction on stochastic context-free languages, see for example [39]. In fact, the only information needed in the sequel is that if structures are modeled by a *consistent *SCFG, then the probability distribution on the production rules of the SCFG implies a probability distribution on the words of the generated language and thus on the modeled structures. To ensure that a SCFG gets consistent, one can for example assign relative frequencies to the productions, which are computed by counting the production rules used in the leftmost derivations of a finite sample of words from the generated language. For unambiguous SCFGs, the relative frequencies can actually be counted efficiently, as for every word, there is only one leftmost derivation to consider.

#### Modeling RNA Secondary Structure via SCFGs

Besides the popular planar graph representation of unknotted secondary structures, many other ways of formalizing RNA folding have been described in literature. One well-established example is the so called *bar-bracket representation*, where a secondary structure is modeled as a string over the alphabet Σ: = {**(**,**)**, **|**}, with a bar **| **and a pair of corresponding brackets **( ) **representing an unpaired nucleotide and two paired bases in the molecule, respectively (see, e.g. [30]). Obviously, both models abstract from primary structure, as they only consider the number of base pairs and unpaired bases and their positions. Moreover, there exists a one-to-one correspondence between both representations, as illustrated by the following example:

**Example 0.1**. The secondary structure shown in Figure 1 has the following equivalent bar-bracket representation that can be decomposed into subwords corresponding to the basic structural motifs that are distinguished in state-of-the-art thermodynamic models:

$$\begin{array}{c} \overset{\text{exterior}\;\text{loop}}{\overset{︷}{\left|||\right|((((\underset{\text{multiloop }(\text{of}\;\text{degree}\;3)}{\underset{︸}{|||hel_{1}|||hel_{2}||hel_{3}|}}))))||,}}\;\text{where}\\ hel_{1}=((((\;\overset{\text{bulge}\;\text{left}}{\overset{︷}{\left|\right|\left|\right|\;(((\;\underset{\text{hairpin}}{\underset{︸}{\left|||||\right|}}\;)))}}\;)))),\;\;hel_{3}=((\;\overset{2\times 2\;\text{interior }\;\text{loop}}{\overset{︷}{\left|\right|\;(\;\underset{2\times 7\;\text{interior }\;\text{loop}}{\underset{︸}{\left|\right|\;((((\;\left||\right|\;))))\;\left||||||\right|}}\;)\;\left|\right|}}\;)),\\ \text{and}\;hel_{2}=(((\;\overset{\text{multiloop }(\text{of}\;\text{degree}\;2)}{\overset{︷}{\left|\right|\;((((\;\underset{1\times 7\;\text{interior }\;\text{loop}}{\underset{︸}{|hel_{2,1}\left||||||\right|}}\;))))\;\left||||\right|hel_{2,2}}}\;))),\text{with}\\ hel_{2,1}=((\;\overset{\text{single}\;\text{bulge}\;\text{left}}{\overset{︷}{|\;(\;\underset{\text{hairpin}}{\underset{︸}{\left|||\right|}}\;)}}\;))\;\text{and}\;hel_{2,2}=(\;\overset{1\times 1\;\text{interior }\;\text{loop}}{\overset{︷}{|((\underset{\text{hairpin}}{\underset{︸}{\left||||\right|}}))|}}\;) \end{array}$$

Note that the reading order of secondary structures is from left to right, which is due to the chemical structure of the molecule.

Consequently, secondary structures without pseudoknots can be encoded as words of a context-free language and the class of all feasible structures can thus effectively be modeled via a corresponding CFG. Basically, that CFG can be constructed to describe a number of classical constraints (e.g. the presence of particular motifs in structures) and it can also express long-range interactions (e.g. base pairings). By extending it to a corresponding SCFG, we can also model the fact that specific motifs of RNA secondary structures are more likely to be folded at certain stages than others (and not all possible motifs are equiprobable at any folding stage).

In fact, it is known for a long time that SCFGs can be used to model RNA secondary structures (see e.g. [40]). Additionally, SCFGs have already been used successfully for the prediction of RNA secondary structure [14,15]. Moreoever, they can be employed for identifying structural motifs as well as for deriving stochastic RNA models that are - with respect to the expected shapes - more realistic than other models [36]. Furthermore, note that an SCFG mirror of the famous Turner energy model has been used in [21] to perform the first analytical analysis of the free energy of RNA secondary structures; this SCFG marks a cornerstone between stochastic and pyhsics-based approaches towards RNA structure prediction.

#### Random Generation With SCFGs

SCFGs can easily be used for the random generation of combinatorial objects according to the probability distribution induced by a sample set, where the only problem is that they do not allow the user to fix the length of generated structures. In particular, given an SCFG  and the corresponding language (combinatorial class) L(G), a random word w∈L(G) can be generated in the following way:

• Start with the sentential form *S *(where *S *denotes the axiom of the grammar ).

• While there are non-terminal symbols (in the currently considered sentential form), do the following:

1) Let *A *denote the leftmost non-terminal symbol.

2) Draw a random number *r *from the interval (0,1].

3) Substitute symbol *A *by the right-hand side *α *of the production *A *→ *α *determined by the random number *r*.

This means consider all *m *≥ 1 rules *p*_1 _: *A *→ *α*_1_,..., *p*_*m *_: *A *→ *α*_*m *_having left-hand side *A*, where according to the definition of SCFGs, $\sum_{i=1}^{m}p_{i}=1$ must hold. Then, find *k *≥ 1 with $\sum_{i=1}^{k-1}p_{i}<r\leq \sum_{i=1}^{k}p_{i}$, i.e. determine *k *≥ 1 with $r\in \left(\sum_{i=1}^{k-1}p_{i},\sum_{i=1}^{k}p_{i}\right]$. The production corresponding to the randomly drawn number *r *∈ (0,1] is then given by *A *→ *α*_*k *_and hence, in the currently considered sentential form, the non-terminal symbol *A *is substituted by *α*_*k*_.

• If there are no more non-terminal symbols, then the currently considered sentential form is equal to a word w∈L(G);w has been randomly generated.

Note that the choice of the production made in 3) according to the previously drawn random number is appropriate, since it is conform to the probability distribution on the grammar rules.

**Example 0.2**. Consider the language generated by the SCFG with productions ¾: *S *→ *ϵ *and ¼: *S *→ (*S*). Thus, we start with the sentential form *S*, then consider the leftmost non-terminal symbol, which is given by *S*, and draw a random number *r *∈ (0,1]. If 0 <*r *≤ ¾, the production determined by *r *is *S *→ *ϵ *and thus, we get the empty word and are finished. Otherwise, ¾ <*r *≤ ¾ + ¼ = 1, which means we have to consider *A *→ (S) for the substitution in step 3) and thus obtain the sentential form (*S*). Afterwards, we must repeat the process, as there is still one non-terminal symbol left.

Unfortunately, there is one major problem that comes with this approach for the (non-uniform) random generation of combinatorial objects: The underlying (consistent) SCFG  implies a probability distribution on the whole language L(G), such that we generate a word of arbitrary size. In order to fix the size, we can proceed along the following lines:

1) We translate the grammar  into a new framework which allows to consider fixed sizes for the random generation, such that

2) the distribution implied on L(G) conditioned on any fixed size *n *is kept within the new framework.

A well-known approach which allows for 1) is connected to the concept of admissible constructions used to describe a decomposable combinatorial class (see above). As the operations (like cartesian products, unions, and so on) used to construct the combinatorial objects are also used to define an order on them, it becomes possible to identify the *i*th object of a given size and the problem of generating objects uniformly at random reduces to the problem of unranking, that is the problem of constructing the object of order (rank) *i*, for *i *a random number (see e.g. [41]).

*Remark*. Some might think that with an appropriate SCFG (modeling a given class of objects) at hand, it is not really necessary to use an unranking method that implies cumbersome formalities such as admissible constructions and decomposable classes if we want to generate random objects of a fixed size *n*. As a matter of principle, they are right - we could also use a *conditional sampling *method: If we need to generate a word of size *n *from non-terminal symbol *A*, where there are *m *≥ 1 rules *f*_*i *_= *A *→ *α*_*i*_, 1 ≤ *i *≤ *m*, having left-hand side *A*, then we just need to choose the next production *f*_*i *_according to

$$\frac{\text{Prob}\left(A\to \alpha_{i}\Rightarrow^{*}x|\text{size}\left(x\right)=n\right)}{\text{Prob}\left(A\Rightarrow^{*}x|\text{size}\left(x\right)=n\right)},$$

which is the posterior probability that we used production rule *f*_*i *_under the condition that a word of size *n *is generated.

Similarly, if the production rule is of the type *A *→ *BC *(assuming the grammar is in Chomsky normal form (CNF), which does not pose a problem, as an unambiguous SCFG can be efficiently transformed into CNF [39]), we can choose a way to split size *n *into sizes *j *and *n - j *for the lengths generated from non-terminal symbols *B *and *C*. This requires precomputing *n length-dependent *probabilities (i.e. all probabilities for generating a word of any length up to *n*) for each non-terminal symbol, which might seem to be similar (with respect to complexity) to precomputing all class sizes up to *n *for all considered combinatorial (sub)classes as needs to be done for unranking. However, there is a striking difference between the two approaches: While conditional sampling makes heavy use of rather small floating point values - with all the well-known problems and discomforting details like underflows or using logarithms associated with it - our unranking approach builds on integer values only which we assume a major advantage. There is another striking difference: length-dependent probabilities (which by the way yield a so-called length-dependent SCFG (LSCFG), see [42], and already have been used in [43]), require a very rich training set. In fact, if the RNA data set used for determining the distribution induced by the grammar is not rich enough, then the corresponding stochastic RNA model is underestimated and its quality decreases. This is especially a problem when considering comprehensive CFGs that distinguish between many different structural motifs in order to get a realistic picture of the molecules' behaviour; such a grammar should however be preferred over simple lightweight grammars as basis for a non-uniform random generation method. Nevertheless, this problem does not surface when sticking to conventional probabilities and the corresponding traditional SCFG model. Actually, since we consider a huge CFG where all possible structural motifs are created by distinct productions, we generally obtain realistic probability distributions and RNA models (see [21]).

Finally note that of course we could make use of random sampling strategies originally designed to sample structures connected to a given sequence in order to generate a random secondary structure only. However, such algorithms typically use a linear time to sample a single base pair (see, e.g., [6]) such that the time to sample a complete structure is quadratic in its length. This causes no problems for the original application of such algorithms since the sequence-dependent preprocessing which is part of their overall procedure is at least quadratic in time and thus the dominating part. Here our approach is of advantage (replacing a factor *n *by *log*(*n*)) and since our preprocessing only depends on the size of the structure to be generated it is performed once and stored to disk for later reuse. Last but not least we are not sure, if the different existing approaches just mentioned could easily be made as fast as ours by simple changes only.

Bottom line is that hooking up to unranking of combinatorial classes offers three significant benefit compared to conditional sampling, namely a fast sampling strategy, the usage of integers instead of floating point values and a greater independence of the richness of the training data (compared to length-dependent models). For this reason, we assume our unranking algorithm a valuable contribution, even though it requires a more cumbersome framework.

### Unranking of Combinatorial Objects

The problem of unranking can easily be solved along the composition of the objects at hand, i.e. the operations used for its construction, once we know the number of possible choices for each substructure. Assume for example we want to unrank objects from a class C=A+B. We will assume all elements of  to be of smaller order than those of  (this way we use the construction of the class to imply an ordering). Finding the *i*th element of , i.e. unranking class , now becomes possible by deciding whether i<card(A). In this case, we recursively call the unranking procedure for . Otherwise (i.e. if i≥card(A)), we consider , searching for its (i-card(A)))th element.

Formally, we first need to specify an order on all objects of the considered combinatorial class that have the same size. This can be done in a recursive way according to the admissible specification of the class:

**Definition 0.6 **( [37]). Neutral and atomic classes contain only one element, such that there is only one possible ordering. Furthermore, let ${<}_{C^{n}}$ denote the ordering within the combinatorial class $C^{n}$, then

• If $C=A_{1}+...+A_{k}$ and $\gamma ,\gamma^{\prime }\in C^{n}$, then $\gamma {<}_{C^{n}}\gamma^{\prime }$ iff

$$\left[\gamma \in {\left(A_{i}\right)}^{n}\;\text{and}\;\gamma^{\prime }\in {\left(A_{j}\right)}^{n}\;\text{and}\;i<j\right]\;\text{or}\;\left[\gamma ,\gamma^{\prime }\in {\left(A_{i}\right)}^{n}\;\text{and}\;\gamma {<}_{{\left(A_{i}\right)}^{n}}\gamma^{\prime }\right].$$

• If C=A×B and $\gamma =\left(\alpha ,\beta \right),\gamma^{\prime }=\left(\alpha^{\prime },\beta^{\prime }\right)\in C^{n}$, then $\gamma {<}_{C^{n}}\gamma^{\prime }$ iff

$$\left[\text{size}\left(\alpha \right)<\text{size}\left(\alpha^{\prime }\right)\right]\text{or}\left[j=\text{size}\left(\alpha \right)=\text{size}\left(\alpha^{\prime }\right)\;\text{and}\;\alpha {<}_{{\left(A\right)}^{j}}\alpha^{\prime }\right]\text{or}\left[\alpha =\alpha^{\prime }\text{and}\;\beta {<}_{\left(B\right)n-j}\beta^{\prime }\right]$$

when considering the lexicographic order (1, 2, 3,..., *n*), which is induced by the specification $C^{n}=A^{0}\times B^{n}+A^{1}\times B^{n-1}+A^{2}\times B^{n-2}+...+A^{n}\times B^{0}$.

• If C=A×B and $\gamma =\left(\alpha ,\beta \right),\gamma^{\prime }=\left(\alpha^{\prime },\beta^{\prime }\right)\in C^{n}$, then $\gamma \gamma {<}_{C^{n}}\gamma^{\prime }$ iff

$$\begin{array}{c} \left[\min\left(\text{size}\left(\alpha \right),\text{size}\left(\beta \right)\right)<\min\left(\text{size}\left(\alpha^{\prime }\right),\text{size}\left(\beta^{\prime }\right)\right)\right]\text{or}\\ \left[\min\left(\text{size}\left(\alpha \right),\text{size}\left(\beta \right)\right)=\min\left(\text{size}\left(\alpha^{\prime }\right),\text{size}\left(B^{\prime }\right)\right)\;\text{and}\;\text{size}\left(\alpha \right)<\text{size}\left(\alpha^{\prime }\right)\right]\text{or}\\ \left[j=\text{size}\left(\alpha \right)=\text{size}\left(\alpha^{\prime }\right)\;\text{and}\;\alpha {<}_{{\left(A\right)}^{j}}\alpha^{\prime }\right]\text{or}\left[\alpha =\alpha^{\prime }\;\text{and}\;\beta {<}_{{\left(B\right)}^{n-j}}\beta^{\prime }\right] \end{array}$$

when considering the boustrophedon order $\left(1,n,2,n-1,...,\left\lceil \frac{n}{2}\right\rceil \right)$, induced by the specification $C^{n}=A^{0}\times B^{n}+A^{n}\times B^{0}+A^{1}\times B^{n-1}+A^{n-1}\times B^{1}+...$

Considering ${<}_{C^{n}}$, the actual unranking algorithms are quite straightforward. Therefore, they will not be presented here and we refer to [20,44] for details.

Recall that in [20], the basic approach towards non-uniform random generation is weighting of combinatorial classes, as this makes it possible that the classes are non-uniformly distributed. If those combinatorial classes are to correspond to a considered SCFG, we have to face the problem that the maximum likelihood (ML) training introduces rational weights for the production rules while weighting as an admissible construction needs integer arguments.

When translating rational probabilities into integral weights, we have to assure that the relative weight of each (unambiguously) generated word remains unchanged. This can be reached by scaling all productions by the same factor (common denominator of all probabilities), while ensuring that derivations are of equal length for words of the same size (ensured by using grammars in CNF). However, a much more elegant way is to scale each production according to its contribution to the length of the word generated, that is, productions lengthening the word by *k *will be scaled by *c*^*k*^. Since we consider CFGs, the lengthening of a production of the form *A *→ *α *is given by |*α*| - 1. However, this rule leads to productions with a conclusion of length 1 not being reweighted, hence we have to assure that all those productions already have integral weights. Furthermore, *ϵ*-productions need a special treatment. We don't want to discuss full details here and conclude by noticing that the *reweighting normal form (RNF) *keeps track of all possible issues:

**Definition 0.7 **( [20]). If $G=\left(I,T,R,S,W\right)$ is a WCFG,  is said to be in *reweighting normal form (RNF) *iff

1.  is loop-free and *ϵ*-free.

2. For all *A *→ *α *∈ *R *with *A *= *S*, we have |*α*| ≤ 1.

3. For all *A *→ *α *∈ *R *with *A *≠ *S*, we have |*α*| > 1 or *W*(*A *→ *α*) ∈ ℕ.

4. For *all A *∈ *I *there exists *α *∈ (*I *∪ *T*)* such that *A *→ *α *∈ *R*.

Note that the last condition (that any intermediate symbol occurs as premise of at least one production) is not required for reweighting, but necessary for the translation of a grammar into an admissible specification.

**Definition 0.8 **( [20]). A WCFG  is called *loop-free *iff there exists no nonempty derivation *A *⇒^+ ^*A *for *A *∈ *I*. It is called *ϵ*-*free *iff there exists no (*A*, *ϵ*) ∈ *R *with *A *= *S *and there exists no (*A*, *α*_1_*Sα*_2_) ∈ *R*, where *ϵ *denotes the empty word.

If  and $G^{\prime }$ are WCFGs, then  and $G^{\prime }$ are said to be *word-equivalent *iff $L\left(G\right)=L\left(G^{\prime }\right)$ and for each word w∈L(G), we have *W*(*w*) = *W*'(*w*).

In [20], it is shown how to transform an arbitrary WCFG to a word-equivalent, loop-free and *ϵ*-free grammar, that grammar to one in RNF and the latter to the corresponding admissible specification. Formally:

**Theorem 0.1 **( [39]). *If **is a SCFG, there exists a SCFG *$G^{\prime }$*in Chomsky normal form (CNF) that is word-equivalent to *, *and *$G^{\prime }$*can be effectively constructed from *.

The construction given in [39] assumes that  is *ϵ*-free. It can however be extended to non-*ϵ*-free grammars by adding an additional step after the intermediate grammar  has been created (see e.g. [20]). Furthermore, it should be noted that an unambiguous grammar is inevitably loop-free.

**Theorem 0.2 **( [20]). *If **is a loop-free*, *ϵ*-*free WCFG, there exists a WCFG *$G^{\prime }$*in RNF that is word-equivalent to **and *$G^{\prime }$*can be effectively constructed from *.

Altogether, starting with an arbitrary unambiguous SCFG $G_{0}$ that models the class of objects to be randomly generated, we have to proceed along the following lines:

• Transform $G_{0}$ to a corresponding *ϵ*-free and loop-free SCFG $G_{1}$.

• Transform $G_{1}$ into $G_{2}$ in RNF (where all production weights are rational).

• Reweight the production rules of $G_{2}$ (such that all production weights are integral), yielding reweighted WCFG $G_{3}$.

• Transform $G_{3}$ (with integral weights) into the corresponding admissible specification.

• This specification (with weighted classes) can be translated directly

- into a recursion for the function size of all involved combinatorial (sub)classes (where class sizes are weighted) and

- into generating algorithms for the specified (weighted) classes,

yielding the desired weighted unranking algorithm for generating random elements of $L\left(G_{0}\right)$.

A small example that shows how to proceed from SCFG to reweighted normal form and the corresponding weighted combinatorial classes which allow for non-uniform generation by means of unranking is discussed in the Appendix.

## Generating Random RNA Secondary Structures

We will now consider the previously discussed approach to construct a weighted unranking algorithm that generates random RNA secondary structures of a given size according to a realistic probability distribution. As for this paper, the corresponding probability distribution will be induced by a set of sample (SSU and LSU r)RNA secondary structures from the databases [45,46], which will be referred to as *biological database *in the sequel. However, the presented algorithm can easily be used for any other distribution, which can be defined by a database of known RNA structures of a particular RNA type; our webservice implementation accessible at http://wwwagak.cs.uni-kl.de/NonUniRandGen is actually able to sample random secondary structures of any specified RNA type. A link to download an implementation of our algorithm (in Wolfram Mathematica) can be found there, too.

## Considered Combinatorial Class

According to the common definition of RNA secondary structure, we decided to consider the combinatorial class of all RNA secondary structures without pseudoknots that meet the stereochemical constraint of hairpin loops consisting of at least 3 unpaired nucleotides, formally:

**Definition 0.9 **( [21]). The language  containing exactly all RNA secondary structures is given by (note that according to this definition, completely unpaired structures are prohibited) $L:=L_{u}L_{lu}^{+}$, where ${ℒ}_{lu}:=({ℒ}_{l}){ℒ}_{u},{ℒ}_{u}:={\left\{|\right\}}^{*}$ is the language of all bar-bracket representations of single-stranded regions and $L_{l}$ is the language of all bar-bracket representations of other possible substructures, i.e. is the smallest language satisfying the following conditions:

1. ${\left\{|\right\}}^{+}\backslash \left\{\left|,|\right|\right\}\subset L_{l}$ (bar-bracket representations of *hairpin loops*).

2. If $w\in L_{l}$, then $(w)\in {ℒ}_{l}$ (bar-bracket representation of a *stacked pair*).

3. If $w\in L_{l}$, then ${\left\{|\right\}}^{+}\left(w\right)\subset L_{l}$ and $(w){\left\{|\right\}}^{+}\subset {ℒ}_{l}$ (bar-bracket representations of *bulge loops*).

4. If $w\in L_{l}$, then ${\left\{|\right\}}^{+}\left(w\right){\left\{|\right\}}^{+}\subset L_{l}$ (bar-bracket representations of *interior loops*).

5. If $w_{1},...,w_{n}\in L_{l}$ and *n *≥ 2, then $L_{u}\left(w_{1}\right)L_{u}\left(w_{2}\right)\cdots L_{u}\left(w_{n}\right)L_{u}\subset L_{l}$ (bar-bracket representations of *multibranched loops*).

The desired weighted unranking algorithm thus generates, for a given size *n *and a given number $i\in \left\{0,...,\text{card}\left(L^{n}\right)-1\right\}$, the *i*th secondary structure $s\in L^{n}$, where $\text{card}\left(L^{n}\right)=\text{size}\left(L,n\right)$ is the number of elements in the weighted class $L^{n}$.

## Considered SCFG Model

First, we have to find a suitable SCFG that generates  and models the distribution of the sample data as closely as possible. To reach this goal, it is important to appropriately specify the set of production rules in order to guarantee that all substructures that have to be distinguished are generated by different rules. This is due to the fact that by using only one production rule *f *to generate different substructures (e.g. any unpaired nucleotides independent of the type of loop they belong to), there is only one weight (the probability *p*_*f *_of this production *f*) with which any of these substructures is generated, whereas the use of different rules *f*_1_,..., *f*_*k *_to distinguish between these substructures implies that they may be generated with different probabilities ${p_{f}}_{{}_{1}},...p_{f_{k}}$, where $p_{f_{1}}+...+p_{f_{k}}=p_{f}$. This way, we ensure that more common substructures are generated with higher probabilities than less common ones.

**Example 0.3**. A (rather simple) unambiguous SCFG $G_{s}$ generating the language  is given by:

$$\begin{array}{cccc} w_{1}: & S_{s}\to CA,\\ w_{2}: & A\to \left(B\right)C, & w_{3}: & A\to \left(B\right)CA,\\ w_{4}: & B\to |||C, & w_{5}: & B\to CA,\\ w_{6}: & C\to \epsilon , & w_{7}: & C\to |C. \end{array}$$

This grammar unambiguously generates  for the following reasons:

• Every sentential form *C***(***B***)***C***(***B***) **... **(***B***)***C *obviously is generated in a unique way; this resembles $L=L_{u}L_{lu}^{+}$ and ${ℒ}_{lu}:=({ℒ}_{l}){ℒ}_{u}$ of L′s definition. The number of outermost pairs of brackets in the entire string uniquely determines the corresponding sentential form to be used.

• Now *B *either generates a hairpin-loop **|**^≥3^, which unambiguously is possible by rules *B *→ **|||***C*, *C *→ **|***C *and *C *→ *ϵ*, or

• *B *itself has to generate at least one additional pair of brackets. In this case, *B *→ *CA *must be applied (only *A *can generate brackets) and then *A *→ **(***B***)***C *resp. *A *→ **(***B***)***CA *are used; the number of outermost brackets to be generated (from *B *under consideration) again uniquely determines that part of the derivation.

When changing the production *w*_5_: *B *→ *CA *used to generate any possible *k*-loop for *k *≥ 2 (any loop that is not a hairpin loop) with probability *w*_5 _into the two rules

$$w_{5.1}:B\to C\left(B\right)C,\;w_{5.2}:B\to C\left(B\right)CA,$$

where *w*_5.1 _+ *w*_5.2 _= *w*_5_, it becomes possible to generate any possible 2-loop (i.e. a stacked pair, a bulge (on the left or on the right), or an interior loop) and all kinds of multiloops (i.e. any *k*-loop with *k *≥ 3) with different probabilities, which could increase the accuracy of the SCFG model. By additionally replacing the first of these two new rules, *w*_5.1 _: *B *→ *C***(***B***)***C*, by the four productions

$$w_{5.1.1}\;:\;B\to \left(B\right),\;\;w_{5.1.2}\;:\;B\to \left|C\left(B\right),\;\;w_{5.1.3}\;:\;B\to \left(B\right)C\right|,\;\;w_{5.1.4}\;:\;B\to \left|C\left(B\right)C\right|,$$

where (*w*_5.1.1 _+ ... + *w*_5.1.4_) + *w*_5.2 _= *w*_5.1 _+ *w*_5.2 _= *w*_5_, we can distinguish between the different types of 2-loops more accurately, yielding a more realistic secondary structure model. In fact, in the case of significant differences of the new probabilities (*w*_5.1.1_, ..., *w*_5.1.4 _and *w*_5.2_), we can expect a huge improvement in the model's accuracy. Note that it is not hard to see that changes to a grammar like the ones just discussed do not change the language generated. However, this is not at all obvious with respect to ambiguity of the grammar.

According to the previously mentioned facts (and the corresponding illustrations by Example 0.3), we decided that the basis for our weighted unranking algorithm should be the following e-free, loop-free and unambiguous (note that these are exactly the preliminary required conditions for the basis SCFG according to [20]) SCFG, which has been derived from the sophisticated SCFG presented in [21] that distinguishes between all known structural motifs that can be found in RNA secondary structure:

**Definition 0.10**. The unambiguous *ϵ*-free SCFG ${\hat{G}}_{\text{sto}}$ generating exactly the language  is given by ${Ĝ}_{\text{sto}}=\left(I_{{Ĝ}_{\text{sto}}},\sum_{{Ĝ}_{\text{sto}}},R_{{Ĝ}_{\text{sto}}},S^{\prime }\right)$, where

$$I_{{\hat{G}}_{\text{sto}}}=\left\{S^{\prime },E,S,T,C,A,L,G,D,B,F,H,P,Q,R,V,W,O,J,K,M,X,Y,Z,N,U\right\},$$

$\sum_{{\hat{G}}_{\text{sto}}}=\{(,),|\}$ and $R_{{Ĝ}_{\text{sto}}}$ contains exactly the following rules:

$$\begin{array}{c} \left(\begin{array}{c} {\hat{p}}_{1}:S^{\prime }\to E,\\ {\hat{p}}_{2}:E\to S,\;{\hat{p}}_{3}:E\to SC,\\ {\hat{p}}_{4}:S\to A,\;{\hat{p}}_{5}:S\to TA,\\ {\hat{p}}_{6}:T\to E,\;{\hat{p}}_{7}:T\to C, \end{array}\right\}\text{shape}\;\text{of}\;\text{exterior}\;\text{loop}\\ {\hat{p}}_{8}:C\to \;\;|,\;\;\;\;{\hat{p}}_{9}:C\to C|,\;\;⇝\text{strands}\;\text{in}\;\text{exterior}\;\text{loop}\\ {\hat{p}}_{10}:A\to \left(L\right),\;\;⇝\text{initiate}\;\text{helix}\\ {\hat{p}}_{11}:L\to A,\;\;{\hat{p}}_{12}:L\to M,\;\;⇝\text{initiate}\;\text{stacked}\;\text{pair}\;\text{or}\;\text{multiple}\;\text{loop}\\ {\hat{p}}_{13}:L\to P,\;\;{\hat{p}}_{14}:L\to Q,\;\;{\hat{p}}_{15}:L\to R,\;\;⇝\text{initiate}\;\text{interior}\;\text{loop}\\ {\hat{p}}_{16}:L\to F,\;\;{\hat{p}}_{17}:L\to G,\;\;⇝\;\;\text{initiate}\;\text{hairpin}\;\text{loop}\;\text{or}\;\text{bulge}\;\text{loop}\\ {\hat{p}}_{18}:G\to A|,\;\;{\hat{p}}_{19}:G\to AD,\;\;{\hat{p}}_{20}:G\to \;\;|A,\;\;{\hat{p}}_{21}:G\to DA,\;\;⇝\text{shape}\;\text{of}\;\text{bulge}\;\text{loop}\\ \left(\begin{array}{c} {\hat{p}}_{22}:D\to B|,\\ {\hat{p}}_{23}:B\to \;|,\;\;\;{\hat{p}}_{24}:B\to B|, \end{array}\right\}\text{strands}\;\text{in}\;\text{bulge}\;\text{loop}\\ \left(\begin{array}{c} {\hat{p}}_{25}:F\to \;|||,\;\;{\hat{p}}_{26}:F\to \;\left|||\right|,\;\;{\hat{p}}_{27}:F\to \;\left|||\right|H,\\ {\hat{p}}_{28}:H\to \;\;|,\;\;{\hat{p}}_{29}:H\to H|, \end{array}\right\}\text{hairpin}\;\text{loop}\\ {\hat{p}}_{30}:P\to \;|A|,\;\;{\hat{p}}_{31}:P\to \;|A||,\;\;{\hat{p}}_{32}:P\to \;||A|,\;\;{\hat{p}}_{33}:P\to \;||A\left|\right|,\;⇝\text{small}\;\text{interior}\;\text{loops}\\ \left(\begin{array}{c} {\hat{p}}_{34}:Q\to \;||O\left|\right|,\;\;{\hat{p}}_{35}:Q\to \;||V|,\\ {\hat{p}}_{36}:R\to \;|O\left|\right|,\;\;{\hat{p}}_{37}:R\to \;\left|\right|W|,\\ {\hat{p}}_{38}:V\to \;JO,\\ {\hat{p}}_{39}:W\to JA,\\ {\hat{p}}_{40}:O\to AK, \end{array}\right\}\text{other}\;\text{interior}\;\text{loops}\\ \left(\begin{array}{c} {\hat{p}}_{41}:J\to \;|,\;\;{\hat{p}}_{42}:J\to J|,\\ {\hat{p}}_{43}:K\to \;|,\;\;{\hat{p}}_{44}:K\to K|, \end{array}\right\}\text{strands}\;\text{in}\;\text{interior}\;\text{loop}\\ \left(\begin{array}{c} {\hat{p}}_{46}:M\to \;XY,\\ {\hat{p}}_{46}:X\to \;A,\;\;{\hat{p}}_{47}:X\to UA,\\ {\hat{p}}_{48}:Y\to \;Z,\\ {\hat{p}}_{49}:Z\to \;X,\;\;{\hat{p}}_{50}:Z\to XN,\\ {\hat{p}}_{51}:N\to \;Z,\;\;{\hat{p}}_{52}:N\to U, \end{array}\right\}\text{multiple}\;\text{loop}\\ {\hat{p}}_{53}:U\to \;|,\;\;{\hat{p}}_{54}:U\to U|,\;⇝\;\;\text{strands}\;\text{in}\;\text{multiple}\;\text{loop} \end{array}$$

**Figure 2:**
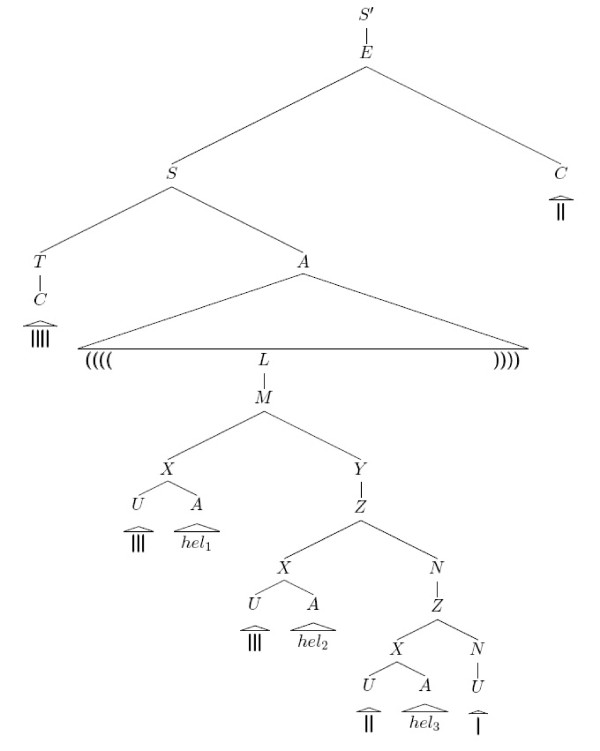
**Unique parse tree for the bar-bracket word considered in Example 0.1 that corresponds to the planar secondary structure from Figure 1**.

**Figure 3:**
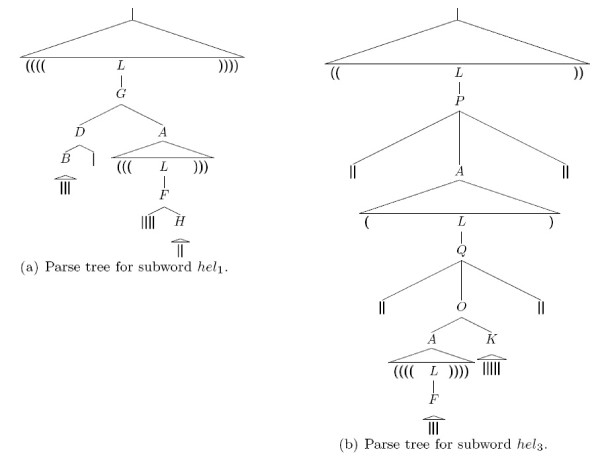
**Particular subtrees of the tree presented in Figure 2**.

Figures 2 and 3 illustrate by examples how (parts of) secondary structures are generated by this SCFG, where we used  to denote the full parse tree for *I *⇒* *x *(i.e. for consecutive applications of an arbitrary number of production rules that generate the subword *x *from the intermediate symbol *I*) in oder to obtain a more compact tree representation. In fact, it is easy to see that the overall structure is always produced by starting with the axiom *S*', while any particular substructure or structural motif that belongs to the combinatorial (sub)class  is created from the corresponding intermediate symbol *I*.

For our application it is crucial that ${Ĝ}_{\text{sto}}$ - as claimed its definition - is unambiguous. To prove this, we first note that ${Ĝ}_{\text{sto}}$ has been constructed starting from a simple grammar which generates  by iteratively replacing one production by several ones (like we did in the previous example) in order to distinguish more and more structural motifs but without changing the language generated. Furthermore, a standard construction to make the grammar *ϵ*-free has been applied. That way, we can be sure that ${Ĝ}_{\text{sto}}$ generates  (formally this fact easily follows by obvious bi-simulation proofs for each substitution and by the proven correctness of the used construction to ensure *ϵ*-freeness). To prove unambiguity, we translate ${Ĝ}_{\text{sto}}$ into a system of equations for its structure generating function (see [47] for details) $S\left[z\right]=\sum_{w\in L}d\left(w\right)z^{\left|w\right|}$ where *d*(*w*) denotes the number of derivation trees ${Ĝ}_{\text{sto}}$ offers for *w*. Eliminating all but the variable associated with the axiom and simplifying (for this step we made use of Mathematica) yields the single equation

$$-z^{5}+S[z](-1+z)(-1+z(2-S[z](-1+z)z+z^{4}))=0$$

This equation is exactly the one of our grammar $G_{s}$ from Example 0.3 which proves that for all *n *both grammars have the same number of derivation trees for words of size *n*. Knowing that both grammars generate  and that $G_{s}$ is unambiguous, the same can be concluded for ${Ĝ}_{\text{sto}}$.

Note that ${Ĝ}_{\text{sto}}$ contains more production rules (and more different non-terminal symbols) than the SCFG considered in [21], but this new grammar is *ϵ*-free and additionally, the right-hand side of every single production contains at most two non-terminal symbols, such that the resulting unranking algorithm has to consider less cases (i.e. less "else if ( )" cases). For details, see [20] and the Appendix.

Furthermore, it should be mentioned that we decided to assign relative frequencies to the production rules of ${Ĝ}_{\text{sto}}$, since such probabilities can be computed efficiently for unambiguous SCFGs. Moreover, by estimating the probabilities ${\hat{p}}_{i},1\leq i\leq 54$, by their relative frequencies, the resulting grammar ${Ĝ}_{\text{sto}}$ has the consistency property, which means ${Ĝ}_{\text{sto}}$ provides a probability distribution on the language $L\left({Ĝ}_{\text{sto}}\right)=L$. In particular, it is well-known that relative frequencies in our context yield a maximum likelihood (ML) estimator for the rule probabilities and thus a consistent estimator for the parameter set. We have trained the probabilities (relative frequencies) of ${Ĝ}_{\text{sto}}$ from the structures $s\in L\left({Ĝ}_{\text{sto}}\right)$ given in our biological database. The resulting probabilities are given in Table 1, their floating point approximations, rounded to the third decimal place in Table 2.

**Table 1:** The probabilities (relative frequencies) for the production rules of the SCFG ${Ĝ}_{\text{sto}}$ obtained by training it using our biological database

Nonterminal *Nt*	Probabilities of Rules with Premise *Nt*
S'	${\hat{p}}_{1}:=1$
E	${\hat{p}}_{2}:=\frac{137}{6476},\;{\hat{p}}_{3}:=\frac{6339}{6476},$
S	${\hat{p}}_{4}:=\frac{177}{12952},\;{\hat{p}}_{5}:=\frac{12775}{12952},$
T	${\hat{p}}_{6}:=\frac{11086}{12775},\;{\hat{p}}_{7}:=\frac{1689}{12775},$
C	${\hat{p}}_{8}:=\frac{14367}{148978},\;{\hat{p}}_{9}:=\frac{134611}{148978},$
A	${\hat{p}}_{10}:=1,$
L	$\begin{array}{cccc} {\hat{p}}_{11}:=\frac{605069}{792975}, & {\hat{p}}_{12}:=\frac{31912}{792975}, & {\hat{p}}_{13}:=\frac{4912}{264325}, & {\hat{p}}_{14}:=\frac{5821}{158595}, \end{array}$
	$\begin{array}{ccc} {\hat{p}}_{15}:=\frac{1893}{264325}, & {\hat{p}}_{16}:=\frac{2723}{31719}, & {\hat{p}}_{17}:=\frac{38399}{792975}, \end{array}$
G	${\hat{p}}_{18}:=\frac{11667}{38399},\;{\hat{p}}_{19}:=\frac{7235}{38399},\;{\hat{p}}_{20}:=\frac{11831}{38399},\;{\hat{p}}_{21}:=\frac{7666}{38399},$
D	${\hat{p}}_{22}:=1,$
B	${\hat{p}}_{23}:=\frac{4967}{12748},\;{\hat{p}}_{24}:=\frac{7781}{12748},$
F	${\hat{p}}_{25}:=\frac{3912}{68075},\;{\hat{p}}_{26}:=\frac{23208}{68075},\;{\hat{p}}_{27}:=\frac{8191}{13615},$
H	${\hat{p}}_{28}:=\frac{8191}{40700},\;{\hat{p}}_{29}:=\frac{32509}{40700},$
P	${\hat{p}}_{30}:=\frac{533}{4912},\;{\hat{p}}_{31}:=\frac{1053}{4912},\;{\hat{p}}_{32}:=\frac{2963}{14736},\;{\hat{p}}_{33}:=\frac{7015}{14736},$
Q	${\hat{p}}_{34}:=\frac{4986}{29105},\;{\hat{p}}_{35}:=\frac{24119}{29105},$
R	${\hat{p}}_{36}:=\frac{2357}{5679},\;{\hat{p}}_{37}:=\frac{3322}{5679},$
V	${\hat{p}}_{38}:=1,$
W	${\hat{p}}_{39}:=1,$
O	${\hat{p}}_{40}:=1,$
J	${\hat{p}}_{41}:=\frac{27441}{84620},\;{\hat{p}}_{42}:=\frac{57179}{84620},$
K	${\hat{p}}_{43}:=\frac{15731}{53725},\;{\hat{p}}_{44}:=\frac{37994}{53725},$
M	${\hat{p}}_{45}:=1,$
X	${\hat{p}}_{46}:=\frac{6196}{87035},\;{\hat{p}}_{47}:=\frac{80839}{87035},$
Y	${\hat{p}}_{48}:=1,$
Z	${\hat{p}}_{49}:=\frac{2812}{55123},\;{\hat{p}}_{50}:=\frac{52311}{55123},$
N	${\hat{p}}_{51}:=\frac{7737}{17437},\;{\hat{p}}_{52}:=\frac{9700}{17437},$
U	${\hat{p}}_{53}:=\frac{109939}{518817},\;{\hat{p}}_{54}:=\frac{408878}{518817},$

**Table 2:** Floating point approximations of the probabilities (relative frequencies) for the production rules of the SCFG ${Ĝ}_{\text{sto}}$ (rounded to three decimal places)

Nonterminal *Nt*	Probabilities of Rules with Premise *Nt*
S'	${\hat{p}}_{1}:=1.000,$
E	${\hat{p}}_{2}:=0.021,\;{\hat{p}}_{3}:=0.979,$
S	${\hat{p}}_{4}:=0.014,\;{\hat{p}}_{5}:=0.986,$
T	${\hat{p}}_{6}:=0.868,\;{\hat{p}}_{7}:=0.132,$
C	${\hat{p}}_{8}:=0.096,\;{\hat{p}}_{9}:=0.904,$
A	${\hat{p}}_{10}:=1.000$
L	${\hat{p}}_{11}:=0.763,\;{\hat{p}}_{12}:=0.040,\;{\hat{p}}_{13}:=0.019,\;{\hat{p}}_{14}:=0.037,$
	${\hat{p}}_{15}:=0.007,\;{\hat{p}}_{16}:=0.086,\;{\hat{p}}_{17}:=0.048,$
G	${\hat{p}}_{18}:=0.304,\;{\hat{p}}_{19}:=0.188,\;{\hat{p}}_{20}:=0.308,\;{\hat{p}}_{21}:=0.200,$
D	${\hat{p}}_{22}:=1.000$
B	${\hat{p}}_{23}:=0.390,\;{\hat{p}}_{24}:=0.610,$
F	${\hat{p}}_{25}:=0.057,\;{\hat{p}}_{26}:=0.341,\;{\hat{p}}_{27}:=0.602,$
H	${\hat{p}}_{28}:=0.201,\;{\hat{p}}_{29}:=0.799,$
P	${\hat{p}}_{30}:=0.109,\;{\hat{p}}_{31}:=0.214,\;{\hat{p}}_{32}:=0.201,\;{\hat{p}}_{33}:=0.476,$
Q	${\hat{p}}_{34}:=0.171,\;{\hat{p}}_{35}:=0.829,$
R	${\hat{p}}_{36}:=0.415,\;{\hat{p}}_{37}:=0.585,$
V	${\hat{p}}_{38}:=1.000$
W	${\hat{p}}_{39}:=1.000$
O	${\hat{p}}_{40}:=1.000$
J	${\hat{p}}_{41}:=0.324,\;{\hat{p}}_{42}:=0.676,$
K	${\hat{p}}_{43}:=0.293,\;{\hat{p}}_{44}:=0.707,$
M	${\hat{p}}_{45}:=1.0000$
X	${\hat{p}}_{46}:=0.071,\;{\hat{p}}_{47}:=0.929,$
Y	${\hat{p}}_{48}:=1.0000$
Z	${\hat{p}}_{49}:=0.051,\;{\hat{p}}_{50}:=0.949,$
N	${\hat{p}}_{51}:=0.444,\;{\hat{p}}_{52}:=0.556,$
U	${\hat{p}}_{53}:=0.212,\;{\hat{p}}_{54}:=0.788.$

In oder to see if over-fitting is an issue for our sophisticated grammar and its rich parameter set, i.e. to see if our training set is large enough to derive reliable values for the rule probabilities, we performed the following experiments: We selected a random 90% (resp. 50%) portion of the original training set and re-estimated the probabilities of all the grammar rules. This process was iterated 40 times, resulting in a sample of 40 parameter sets. Finally, for each parameter we determined its variance along this sample of size 40. The corresponding values lay between 0 (resulting for intermediate symbols without alternatives; for whose productions a probability of 1 is predetermined) and 2.87652 × 10^-6 ^(resp. 2.86242 × 10^-5^). We can conclude that over-fitting is no issue in connection with our sophisticated grammar and the training set used.

## Derivation of the Algorithm

The elaborate SCFG ${Ĝ}_{\text{sto}}$ is appropriate for being used as the basis for the desired weighed unranking method: after having determined the RNF of this SCFG and the corresponding weighted combinatorial classes, we easily find a recursion for the size function (in the same ways as discussed in Example App-.4). Then, we can use the resulting weighted class sizes for the straightforward construction of the desired unranking algorithm.

In fact, for the construction of the complete algorithm, we simply have to use Algorithms 1 to 4 (Unranking of neutral classes, atomic classes, disjoint unions and cartesian products, respectively) and Algorithm 6 (Unranking of weighted classes) given in [20] as subroutines. However, to improve the worst-case complexity of the resulting unranking procedure from $O\left(n^{2}\right)$ to $O\left(n\cdot \log\left(n\right)\right)$ by using the *boustrophedonic order *instead of the *sequential order*, a simple change in Algorithm 4 (Unranking of cartesian products) is neccessary (see e.g. [7]).

A random RNA secondary structure of size *n *can easily be computed by drawing a random number i∈{0,…, size(L,n)-1} and then unranking the *i*th structure of size *n*. The worst-case runtime complexity of this procedure is equal to that of unranking and is thus given by $O\left(n\cdot \log\left(n\right)\right)$ when using the boustrophedonic order. By repeating this procedure *m *times, a set of *m *(not necessarily distinct) random RNA secondary structures of size *n *can be generated in time $O\left(m\cdot n\cdot \log\left(n\right)\right)$, where a preprocessing time of $O\left(n^{2}\right)$ is required for the computation of all (weighted) class sizes up to input length *n*.

A complete and detailed description of the derivation of our weighted unranking algorithm for (SSU and LSU r)RNA secondary structures can be found in the Appendix, since it is too comprehensive to be presented here and the different steps for its generation correspond to those described in [20].

### Availability of Software

It may be of interest to the reader that this non-uniform random generation algorithm for RNA secondary structures has been implemented as a webservice which is accessible to the scientific community under http://wwwagak.cs.uni-kl.de/NonUniRandGen. Since it is relevant for researchers to have methods available for generating random structures that are realistic for a particular investigation, this webservice is also capable of allowing the user to specify the distribution from which the corresponding structures should be sampled (in the form of a set of secondary structure samples from which the parameters for our grammars are inferred). Furthermore, our Mathematica source code used to implement the webservice can be downloaded from our website and used under GNU public licence.

## Discussion

The purpose of this section is to analyze the quality of randomly generated structures by considering some experimental results.

### Parameters for Structural Motifs

As a first step, we decided to consider several important parameters related to particular structural motifs of RNA secondary structure and compare the observed statistical values derived from a native sample (here our biological database, i.e. the set of real-life RNA data that we used for deriving the distribution and thus the weights for the unranking algorithm) to those derived from a corresponding random sample (i.e. a set of random structures generated by our algorithm). In order to obtain an appropriate random sample, we have generated exactly one random structure of size *n *for each native RNA structure of size *n *given in our database, such that for each occurring size *n*, the random sample and the native sample contain the same number of structures having this size.

The determined results are presented in Table 3. Comparing the specific values of all different parameters, we can guess that our algorithm produces random RNA secondary structures that are, related to the different structural motifs and thus related to the expected shape of such structures, in most cases realistic. Obviously, this is a major improvement over existing approaches for the random generation of secondary structures of a given input size *n *(where the corresponding specific RNA sequence is *not *known, but only its length *n*), as those (sequence-independent) methods are only capable of generating structures uniformly at random for input size *n*. Furthermore, with the SCFG model used here, we have an new model for RNA secondary structures at hand which realistically reflects the structure of an RNA molecule and its basic structural motifs.

**Table 3:** Expectation and variance of important parameters related to particular structural motifs of RNA secondary structure

Parameter	Expected Value		Variance	
	
	Random	Native	Random	Native
num_unp_	848.179	839.956	98964.7	103426.
num_bps_	420.848	424.96	27785.3	31310.9
num_urs_	179.73	181.822	4959.96	5117.47

num_e_	1.	1.	0.	0.
num_h_	36.6983	36.4818	196.935	185.596
num_s_	321.18	324.26	16538.8	19343.4
num_b_	20.6061	20.5782	87.1894	50.3103
num_i_	26.1442	26.538	125.66	194.769
num_m_	16.2197	17.1018	57.8874	41.0261
num_hel_	99.6683	100.7	1549.24	1492.84

unp_e_	106.014	79.8382	4039.69	3897.61
unp_h_	6.93534	6.93188	18.4264	77.464
unp_s_	--	--	--	--
unp_b_	1.9948	1.99596	3.10283	6.87868
unp_i_	7.14617	7.08869	16.5725	31.1197
unp_m_	16.0122	16.2577	87.4906	195.497
unp_hel_	--	--	--	--

bps_e_	9.41479	6.94105	29.1956	6.30949
bps_h_	--	--	--	--
bps_s_	1.	1.	0.	0.
bps_b_	1.	1.	0.	0.
bps_i_	1.	1.	0.	0.
bps_m_	2.68212	2.72734	1.12921	1.21643
bps_hel_	4.22249	4.22006	13.6266	5.52299

Values are derived from a native sample (our biological database) and from a random sample, respectively. num_*x*_denotes the number of occurrences of motif *_x_*in one secondary structure and unp*_x_*(bps_*x*_) denotes the number of accessible unpaired bases (base pairs) in one substructure of type *x*. unp, bps, urs denote unpaired bases, base pairs and unpaired regions, whereas *e*, *h*, *s*, *b*, *i*, *m*, *hel *denote exterior loop, hairpin loop, stacked pair, bulge loop, interior loop, multiloop and helix, respectively.

### Related Free Energies

For further investigation on the accuracy of our random generator, we take on a completely different point of view and consider thermodynamics. The reason behind this idea is that if an RNA secondary structure model induced by a SCFG shows a realistic behaviour (expectation and variance) with respect to minimum free energy, then it is rather likely that our grammar also shows a realistic picture for all the different structural motifs of a molecule's folding (as the free energy of a molecule's structure is defined as the sum of the energy contributions of all its substructures).

Since we do not know the corresponding RNA sequences for the randomly generated structures, we can not use one of the common sequence-dependent thermodynamic models for RNAs. Therefore, we decided to consider both the *static *and *dynamic *free energy models (in the static model, averaged free energy contributions for the distinguished structural motifs are considered which can easily be derived from the training data (by sequence counting). These averaged values actually represent the free energy contributions that have to be added for the respective whole substructures. For the dynamic model, corresponding average values for length-dependent free energy contributions (that depend on the number of unpaired or paired bases within particular substructures) are added for each component (unpaired base or base pair) in the respective motifs, such that in contrast to the static model, substructures of different lengths are assigned different free energy values) defined in [21] for RNA secondary structures with unknown sequence. These models are based on the well-known Turner energy model [22,23] and model parameters have been derived from the same biological database (of SSU and LSU rRNAs) that we consider in this article. In fact, both models have turned out to show a realistic behaviour and can therefore be used to judge the quality of random structures generated by our algorithm.

#### Unquantified Results

Similar to [21], we denote the free energy of a given secondary structure s∈L according to the static and dynamic model by *g*_*stat*_(*s*) and *g*_*dyn*_(*s*), respectively. Moreover, the expected free energy and corresponding variance that have been analytically derived in that paper for any *n *> 0 are denoted by $\mu_{energy,n}:=E\left[energy\left(s\right)|\text{ size}\left(s\right)=n\right]$ and $\sigma_{energy,n}^{2}:=V\left[energy\left(s\right)|\text{ size}\left(s\right)=n\right]$, respectively, where *energy *∈ {*g*_*stat*_*, g*_*dyn*_}. The corresponding confidence interval for *n *> 0 and *k *> 1, which contains at least $\left(100-\frac{100}{k^{2}}\right)$ percent of the energies in $\left\{energy\left(s\right)|s\in L^{n}\right\}$ is denoted by *I*_*energy,n*_(*k*): = (*μ*_*energy,n *_- *k*σ_*energy*_,_n_, *μ*_*energy,n *_+ *k*σ_*energy,n*_). As these analytical energy results from [21] and our unranking algorithm have been derived from the same database of real-life RNA data and by modeling the same class  of structures via very similar SCFGs, it seems adequate to use them for comparisons with the energies of our randomly generated structures.

Before we start with our comparisons, note that for any sample set  of secondary structures, we can calculate the corresponding energy points EP(S,energy):={(size(s),energy(s))∣s∈S}, where *energy *∈ {*g*_*stat*_, *g*_*dyn*_}. Obviously, we can also compute the corresponding "average energy points" $AvEP\left(S,energy\right):=\left\{\left(n,\mu_{n}:=\frac{1}{\text{card}\left(S^{n}\right)}\sum_{s\in S^{n}}energy\left(s\right)\right)|S^{n}\neq \emptyset \right\}$ and the corresponding "energy variance points" $VarEP(S,energy):=\left\{(n,\sigma_{n}^{2}:=\frac{1}{\text{card}(S^{n})}\sum_{s\in S^{n}}{\left(\mu_{n}-energy(s)\right)}^{2}|S^{n}\neq \emptyset \right\}$, respectively. In the sequel, we will denote a random sample generated by our algorithm by ℛ and a native sample (biological database) by .

In order to obtain an appropriate random sample for our energy comparisons, we derived a large set of random structures by generating 1000 RNA secondary structures for each of the sizes *n *∈ {500,1000,1500,..., 5000, 5500} with our weighted unranking algorithm. To compare the energies of our randomly generated structures to the corresponding confidence interval(s), we decided to consider any $k\in \left\{\sqrt{2},2,\sqrt{10},\sqrt{20}\right\}$, meaning the probability that the free energy of a random RNA secondary structure of size *n *lies within the corresponding interval is greater than 0.5, 0.75, 0.9, and 0.95, respectively.

**Figure 4:**
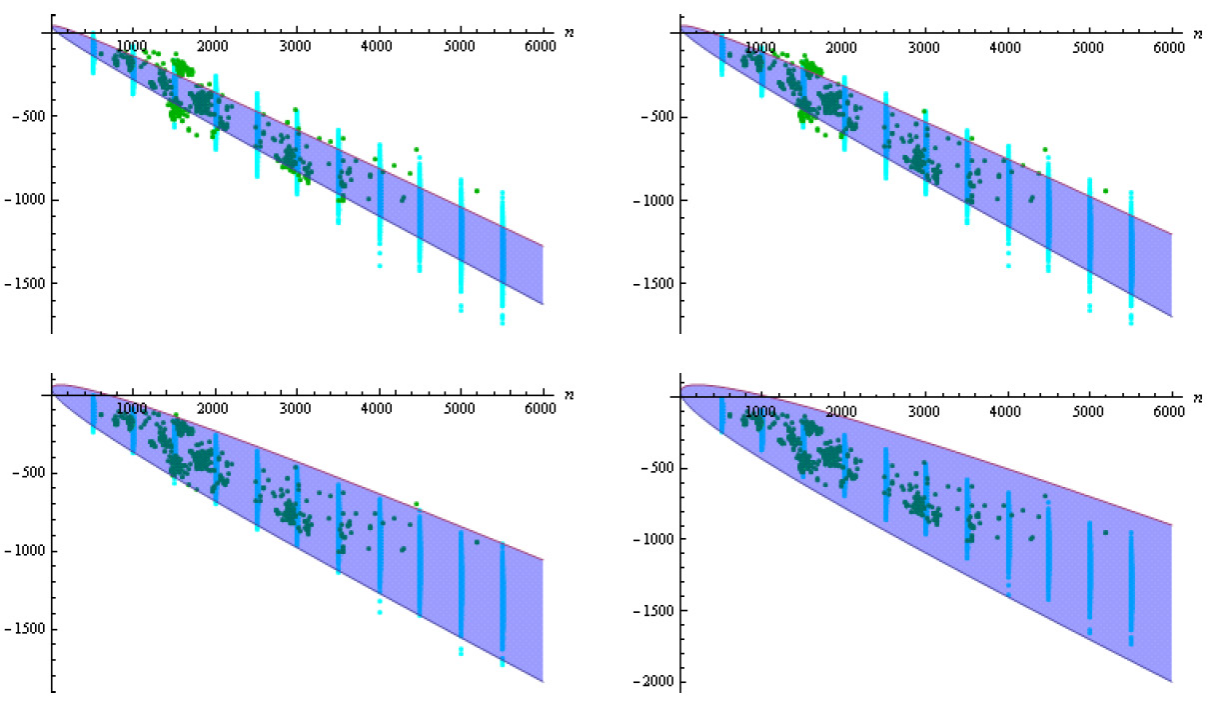
**Plots of the confidence intervals $I_{g_{stat},n\left(k\right)}$**. Intervals are shown for the static energy model (blue), for $k\in \left\{\sqrt{2},2,\sqrt{10},\sqrt{20}\right\}$ (top left to bottom right), together with the corresponding energy points $EP(ℛ,g_{stat})$ for the random sample (cyan) and $EP\left(N,g_{stat}\right)$ for the native sample (green).

**Figure 5:**
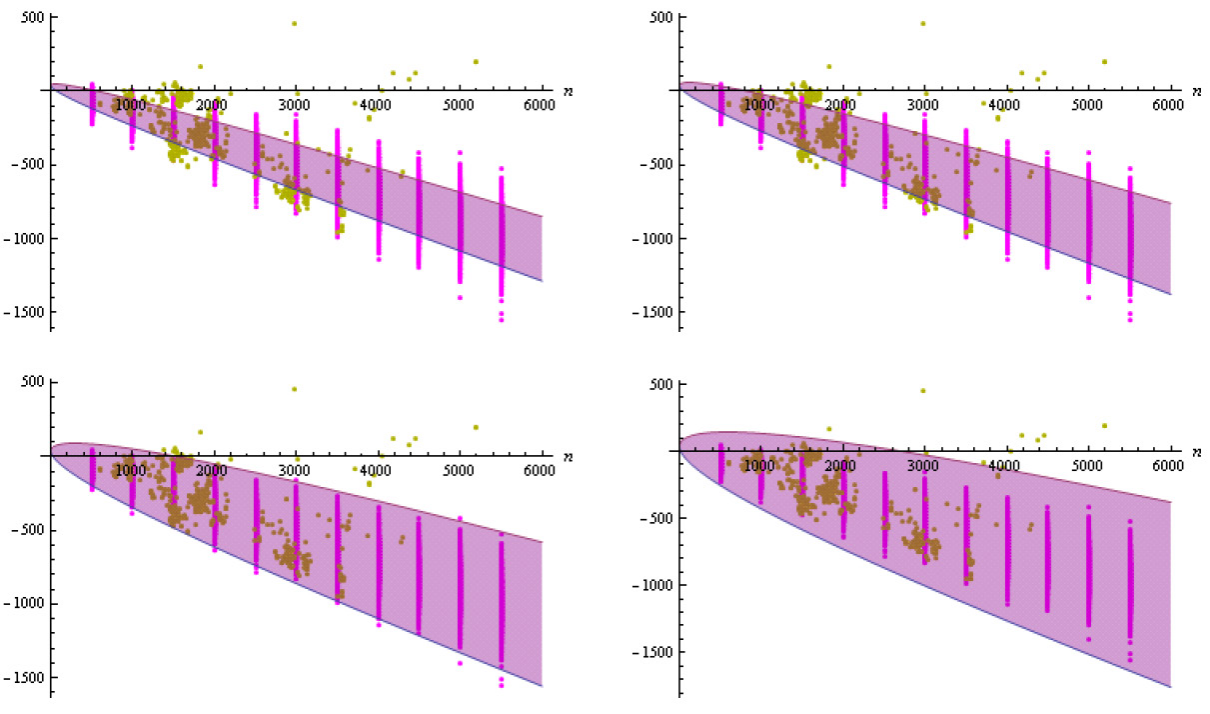
**Plots of the confidence intervals $I_{g_{dyn},n\left(k\right)}$**. Intervals are shown for the dynamic energy model (purple), for $k\in \left\{\sqrt{2},2,\sqrt{10},\sqrt{20}\right\}$ (top left to bottom right), together with the corresponding energy points $EP(ℛ,g_{dyn})$ for the random sample (magenta) and $EP\left(N,g_{dyn}\right)$ for the native sample (yellow).

**Figure 6:**
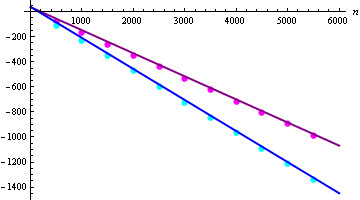
**Plot of expectations of the free energy**. Plots show $\mu_{g_{stat},n}$ (blue) and $\mu_{g_{dyn},n}$ (purple) of a random RNA secondary structure of size *n*, together with the "average energy points" $AvEP(ℛ,g_{stat})$ (cyan) and $AvEP(ℛ,g_{dyn})$ (magenta) for the random sample.

**Figure 7:**
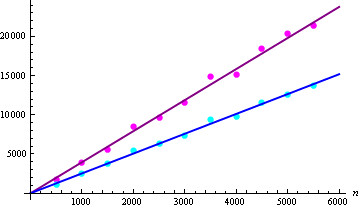
**Plot of variances of the free energy**. Plots show $\sigma_{g_{stat},n}^{2}$ (blue) and $\sigma_{g_{dyn},n}^{2}$ (purple) of a random RNA secondary structure of size *n*, together with the "energy variance points" $VarEP(ℛ,g_{stat})$ (cyan) and $VarEP(ℛ,g_{dyn})$ (magenta) for the random sample.

Figure 4 shows a plot of the corresponding four confidence intervals (analytically derived, related to our biological data) along with the energy points for our random sample and for our native database, respectively, under the assumption of the static energy model. The corresponding plots for the dynamic energy model are shown in Figure 5. Looking at both figures, we immediately see that the energies for our set of randomly generated RNA secondary structures seem to fit to the ones for the considered RNA database and also to the corresponding analytically obtained energy results from [21]. This observation becomes even more clear by considering Figures 6 and 7. There, we compare the previously introduced "average energy points" and "energy variance points" to the analytically determined expected free energy and corresponding variance from [21], respectively.

#### Quantified Results

The previously considered energy comparisons have been presented only by unquantified plots. This may not be very satisfying, since it is obvious that the free energy would decrease with structure size and aside from this, it could have been expected that for large randomly generated sets of structures of a given size, the average energy and corresponding variance fit the analytically obtained energy results derived under the assumption of a basically equivalent SCFG model for secondary structures. Therefore, there is a need to consider some sort of quantification and additionally present corresponding quantified comparison results. What really matters is the degree to which the energy ranges of the random structures agree, in distribution, with our biological database. This means we have to find out if the energies related to a random sample (generated by our unranking method) and those related to a native sample (given by the structures in our biological database) come from a common distribution. Consequently, we have to consider the energies of a random sample and those of a native one as two independent sets of values and determine the extend to which their distributions coincide, or in other words to test for significant differences between these two sets. For this reason, we decided to apply one of the most common (non-parametric) significance tests known from statistics, the so-called *Mann-Whitney U-test *[48], which is widely used as statistical hypothesis test for assessing whether two independent samples of observations (with arbitrary sample sizes) come from the same distribution. It is also known as the *Wilcoxon rank-sum test *[49] which however can only be applied for equal sample sizes.

Formally, this test is used to check whether the *null hypothesis N*_0 _- which states that the two independent samples *X *and *Y *are identically distributed (i.e. *F*(*X*) = *F*(*Y*)) - can be accepted or else, has to be rejected. More specifically, the result of such a test, the so-called *p-value*, is a probability answering the following question: If the two samples really have the same distribution, what is the probability that the observed difference is due to chance alone? In other words, were the deviations (differences between the two samples) the result of chance, or were they due to other factors and how much deviation can occur before one must conclude that something other than chance causes the differences? The *p*-value is called *statistically significant *if it is unlikely that the differences occurred by chance alone, according to a preliminary chosen threshold probability, the *significance level α *(common choices are e.g. *α *∈ {0.10,0.05,0.01}). If *p *≥ *α*, the deviation is small enough that chance alone accounts for it; this is within the range of acceptable deviation. If *p *< α, we must conclude that some factor other than chance causes the deviation to be so great, this will lead us to decide that the two sets come from different distributions.

For our analysis, we again decided to generate the same numbers of random structures for any size as are given for this size in our biological database, such that random and native sample contain the same numbers of structures for any occurring size (and hence the sample sizes are equal). Moreover, note that the unquantified results presented in Figures 4 and 5 might yield the assumption that for any structure size, some energy values of randomly generated structures are scattered too widely around the corresponding expected value, such that those randomly drawn secondary structures can not be considered realistic (neither with respect to thermodynamics nor with respect to structural composition and expected shape). In an attempt to disprove that assumption, we decided to perform a series of Wilcoxon tests by considering a number of different random samples. These samples are created by obeying a specified energy-based rejection scheme: Do not add a randomly generated structure of a given size to the sample if its free energy (according to the static or dynamic model or according to both models) lies outside the corresponding confidence interval(s). Formally, for any preliminary chosen value *k *> 1, a generated structure $s\in L^{n}$ is added to the random sample iff

$$\begin{array}{lll} & \left[g_{stat}\in I_{g_{stat},n}\left(k\right)\text{ (variant “static”)}\right]\text{ or }\left[g_{dyn}\in I_{g_{dyn},n}\left(k\right)\text{ (variant “dynamic”)}\right]\text{ or }\; & \\ & \left[g_{stat}\in I_{g_{stat},n}\left(k\right)\text{ and }g_{dyn}\in I_{g_{dyn},n}\left(k\right)\text{ (variant “both”)}\right];\; & \end{array}$$

otherwise it is rejected. This means we accept only a specified deviation of the energy *energy *(*s*) of the random structure *s *from the corresponding expected free energy *μ*_*energy,n *_and reject structures whose energy differs too much from the expected value. Note that for *k *= ∞ (confidence interval *I*_*energy,n*_(*k*) contains 100 percent of the energies *energy*(*s*) of all $s\in L^{n}$), no structures are rejected. Hence, in this case, the corresponding random sample corresponds to the usual (unrestricted) output of our algorithm.

The Wilcoxon test results for our native sample together with any of a number of random sample sets generated in the previously described restricted manner, respectively, can be found in Table 4. As we can see, the best results are achieved for the unrestricted sample sets, where all free energies of randomly generated structures were allowed during the sample creation process. Moreover, these two results (for the unrestricted case *k *= ∞) are not statistically significant when considering the common significance level *α *= 0.05, that is in both cases, we can assume that the energies of the random structures and those of the biological data follow a common distribution. These observations indicate that our weighted unranking algorithm produces random RNA secondary structures that are - related to the free energy of such structures (in expectation and variation) - in expectation realistic.

**Table 4:** Significance results for statistical hypothesis testing, computed by the Wilcoxon rank-sum method

Chosen Value of *k*	Percent Within Corr. Interval	Models Used for Rejection	Model for Native Energies	Model for Random Energies	Resulting Wilcoxon *p*-Value (approx.)
		Dynamic	Dynamic	Dynamic	0.0008438
$\frac{10}{3\sqrt{11}}\approx 1.00504$	1	Static	Static	Static	1.872·10^-9^
		Both	Dynamic	Dynamic	0.000507
		Both	Static	Static	1.851·10^-10^

		Dynamic	Dynamic	Dynamic	0.001567
$\frac{2\sqrt{5}}{\sqrt{19}}\approx 1.02598$	5	Static	Static	Static	1.454·10^-10^
		Both	Dynamic	Dynamic	0.0002654
		Both	Static	Static	1.009·10^-9^

		Dynamic	Dynamic	Dynamic	0.001374
$\frac{\sqrt{10}}{3}\approx 1.05409$	10	Static	Static	Static	3.526·10^-9^
		Both	Dynamic	Dynamic	0.0004116
		Both	Static	Static	9.018·10^-10^

		Dynamic	Dynamic	Dynamic	0.003618
$\frac{2}{\sqrt{3}}\approx 1.15470$	25	Static	Static	Static	2.530·10^-7^
		Both	Dynamic	Dynamic	0.001228
		Both	Static	Static	1.162·10^-7^

		Dynamic	Dynamic	Dynamic	0.02394
$\sqrt{2}\approx 1.41421$	50	Static	Static	Static	1.278·10^-6^
		Both	Dynamic	Dynamic	0.001389
		Both	Static	Static	1.515 10^-7^

		Dynamic	Dynamic	Dynamic	0.1184
2	75	Static	Static	Static	0.001034
		Both	Dynamic	Dynamic	0.0495
		Both	Static	Static	0.0009445

∞	100	--	Dynamic	Dynamic	0.4007
		--	Static	Static	0.08961

Besides that, it is obvious that the computed *p*-values are much better for the dynamic energy model than for the static one. This underlines the suggestion made in [21] that, although both energy models have been proven to be realistic, due to the more realistic variation of free energies connected to varying loop length, the dynamic model should be used for possible applications. Since at least for the dynamic model, the random data fit very nicely with the native data, we can conclude that structures generated by our non-uniform random generation algorithm behave realistic with respect to free energies and - as the energy of the overall structure is assumed to be equal to the sum of the substructure energies - rather likely also with respect to appearance of the different structural motifs of RNA molecules.

## Conclusion

Altogether, we can finally conclude that the non-uniform random generation method proposed in this article produces appropriate output and may thus be used (for research issues as well as for practical applications) to generate random RNA secondary structures. In fact, for any arbitrary type of (pseudoknot-free) RNA, a corresponding random sampler can be derived in the presented way. Actually, our webservice can be used for generating random secondary structures of any specified type of RNA. It just requires a database of known structures for the respective RNA type as input.

Note that in this work, we abstract from sequence and consider only the structure size as input for our algorithm. Thus, an interesting problem for future research would be to find a way to extend the presented realistic SCFG model to additionally deal with RNA sequence. In fact, this work and especially the considered elaborate SCFG could mark some sort of stepping stone towards new stochastic RNA secondary structure prediction methods realized by statistical random sampling.

## Competing interests

The authors declare that they have no competing interests.

## Authors' contributions

MEN and FW have invented the general framework for the non-uniform random generation. AS and MEN designed the SCFG for RNA secondary structures; MEN proved its unambiguity. AS developed and implemented the algorithms for generating random RNA secondary structures. AS performed all experiments and evaluated the quality of our algorithms. MEN supervised the work and development of ideas. AS drafted the manuscript; a revision and its final version have been prepared by MEN. All authors have read and approved the final manuscript.

## Appendix

### How to Construct a Weighted Unranking Algorithm from a Given SCFG

The purpose of this section is to give a rather small example for applying the construction scheme described in detail in [20] to proceed from an arbitrary SCFG to reweighted normal form (RNF) and then to the corresponding weighted combinatorial classes which allow for non-uniform generation by means of unranking.

**Example App-.4**. Let us consider the SCFG $G_{d}$, which contains the following rules:

$$\begin{array}{cccc} w_{1}: & S\to B,\\ w_{2}: & B\to \left(B\right), & w_{3}: & B\to |C,\\ w_{4}: & C\to \epsilon , & w_{5}: & C\to |C. \end{array}$$

To apply the approach presented in [20] to transform a given SCFG to RNF, the grammar needs to be *ϵ*-free and loop-free. Thus, we first have to transform grammar $G_{d}$ into the following one:

$$\begin{array}{cccc} {\hat{w}}_{1}: & S\to B,\\ {\hat{w}}_{2}: & B\to \left(B\right), & {\hat{w}}_{3}: & B\to C,\\ {\hat{w}}_{4}: & C\to |, & {\hat{w}}_{5}: & C\to |C. \end{array}$$

The transformation of $G_{d}$ into RNF now works as follows: First, we have to gather all possible chains *A *→ *A*_1 _→ *A*_2 _→ ... → *α*, where *A *≠ *S *and |*α*| = 1. These chains are *B *→ *C*, *B *→ *C *→ **| **and C → **|**; the rules *B *→ *C *and *C *→ **| **are then removed. Second, we have to replace each of these chains by a specific new rule. In fact, we have to add *B*^*C*,*ϵ *^→ *C*, *B*^|,*C *^→ | and *C*^|,*ϵ *^→ **| **to the new set of productions. Consequently, our new rule set is now given by

$$\begin{array}{cccccc} {\hat{w}}_{1}: & S\to B,\\ {\hat{w}}_{2}: & B\to \left(B\right),\\ {\hat{w}}_{5}: & C\to |C,\\ 1: & B^{C,\epsilon }\to C, & 1: & B^{|,C}\to |, & 1: & C^{|,\epsilon }\to |. \end{array}$$

Third, for each occurrence of a non-terminal symbol *A *in the conclusion of a production and each previously added new rule $A^{\alpha ,A_{1}A_{2}\ldots }\to \alpha$ corresponding to a chain *A *→ *A*_1 _→ *A*_2 _→ ... → *α*, add a specific new rule. This way, we obtain the following production set:

$$\begin{array}{cccccc} {\hat{w}}_{1}: & S\to B, & {\hat{w}}_{1}\cdot {\hat{w}}_{3}: & S\to B^{C,\epsilon }, & {\hat{w}}_{1}\cdot {\hat{w}}_{3}\cdot {\hat{w}}_{4}: & S\to B^{|,C},\\ {\hat{w}}_{2}: & B\to \left(B\right), & {\hat{w}}_{2}\cdot {\hat{w}}_{3}: & B\to \left(B^{C,\epsilon }\right), & {\hat{w}}_{2}\cdot {\hat{w}}_{3}\cdot {\hat{w}}_{4}: & B\to \left(B^{|,C}\right),\\ {\hat{w}}_{5}: & C\to |C, & {\hat{w}}_{5}\cdot {\hat{w}}_{4}: & C\to |C^{|,\epsilon },\\ 1: & B^{C,\epsilon }\to C, & 1: & B^{|,C}\to |, & 1: & C^{|,\epsilon }\to |. \end{array}$$

Fourth, each intermediate symbol that no longer occurs as premise in any of the productions has to be removed and fifth, each production of the form *S *→ *α*, where *S *is the axiom and |*α*| > 1 has to be changed in a specific way. However, since in our case, there is obviously nothing left to do, the transformation of $G_{d}$ into RNF is finished.

For $G_{d}$ (in RNF), where all production weights are rational, we can determine the common denominator *s *of the weights of productions with premise *S*, as well as the common denominator *c *of the weights of the remaining productions (i.e., of the productions with premise *B *or *C*). Then, the reweighting of the production rules of (the RNF of) $G_{d}$ is done by multiplying the weights of productions with source *S *by *s*, and the weights of the other productions *A *→ *α*, where *A *≠ *S*, by the factor *c*^|*α*|-1^. After that, we obtain the following reweighted grammar $G_{d}^{\prime }$:

$$\begin{array}{cccccc} w_{1}^{\prime }: & S\to B, & w_{2}^{\prime }: & S\to B^{C,\epsilon }, & w_{3}^{\prime }: & S\to B^{|,C},\\ w_{4}^{\prime }: & B\to \left(B\right), & w_{5}^{\prime }: & B\to \left(B^{C,\epsilon }\right), & w_{6}^{\prime }: & B\to \left(B^{|,C}\right),\\ w_{7}^{\prime }: & C\to |C, & w_{8}^{\prime }: & C\to |C^{|,\epsilon },\\ 1: & B^{C,\epsilon }\to C, & 1: & B^{|,C}\to |, & 1: & C^{|,\epsilon }\to |, \end{array}$$

where each $W_{i}^{\prime },\;1\leq i\leq 8$, is integral.

The (now weighted) grammar can easily be translated into a corresponding admissible specification, which includes the weighting of all involved combinatorial (sub)classes, as described earlier. For the reweighted grammar $G_{d}^{\prime }$, this specification is given by the following equations:

$$\begin{array}{ccc} \;\;\;S_{1}=B, & \;\;\;\;\;\;S_{2}=B^{C,\varepsilon }, & \;S_{3}=B^{|,C},\\ \;\;\;B_{1}=Z_{\left(\times B\times Z\right)}, & \;\;\;\;\;\;B_{2}=Z_{\left(\times B^{C,\varepsilon }\times Z\right)}, & \;B_{3}=Z_{\left(\times B^{|,C}\times Z\right)},\\ \;\;\;C_{1}=Z_{|}\times C, & \;\;\;\;\;\;C_{2}=Z_{|}\times C^{|,\varepsilon }, & \\ B^{C,\varepsilon }=C, & \;\;\;\;B^{|,C}=Z_{|}, & C^{|,\varepsilon }=Z_{|},\\ & S=w_{1}^{\prime }\cdot S_{1}+w_{2}^{\prime }\cdot S_{2}+w_{3}^{\prime }\cdot S_{3}, & \\ & B=w_{4}^{\prime }\cdot B_{1}+w_{5}^{\prime }\cdot B_{2}+w_{6}^{\prime }\cdot B_{3}, & \\ & C=w_{7}^{\prime }\cdot C_{1}+w_{8}^{\prime }\cdot C_{2}, & \end{array}$$

which can be simplified in the following way:

$$\begin{array}{lll} {ℬ}_{1}=Z_{\left(\times ℬ\times Z\right)}, & \;\;\;{ℬ}_{2}=Z_{\left(\times C\times Z\right)}, & {ℬ}_{3}=Z_{\left(\times Z_{|}\times Z\right)},\\ C_{1}=Z_{|}\times C, & \;\;\;\;\;\;C_{2}=Z_{|}\times Z_{|}, & \\ & S=w_{1}^{\prime }\cdot ℬ+w_{2}^{\prime }\cdot C+w_{3}^{\prime }\cdot Z_{|}, & \\ & ℬ=w_{4}^{\prime }\cdot {ℬ}_{1}+w_{5}^{\prime }\cdot {ℬ}_{2}+w_{6}^{\prime }\cdot {ℬ}_{3}, & \\ & C=w_{7}^{\prime }\cdot C_{1}+w_{8}^{\prime }\cdot C_{2}. & \end{array}$$

As described earlier, this specification (with weighted classes) derived from reweighted grammar $G_{d}^{\prime }$ transforms immediately into a recursion for the function size of all needed combinatorial classes. For $G_{d}^{\prime }$, the recursion for the function size has the following form:

$$\mathrm{size}\left(I,n\right):=\left\{\begin{array}{cc} \mathrm{size}\left(B,n-2\right)\; & I=B_{1},\\ \mathrm{size}\left(C,n-2\right)\; & I=B_{2},\\ 1\; & I=B_{3}\text{ and }n=3,\\ \mathrm{size}\left(C,n-1\right)\; & I=C_{1},\\ 1\; & I=C_{2}\text{ and }n=2,\\ w_{1}^{\prime }\cdot \mathrm{size}\left(B,n\right)+w_{2}^{\prime }\cdot \mathrm{size}\left(C,n\right)+w_{3}^{\prime }\cdot 1\; & I=S\text{ and }n=1,\\ w_{1}^{\prime }\cdot \mathrm{size}\left(B,n\right)+w_{2}^{\prime }\cdot \mathrm{size}\left(C,n\right)+w_{3}^{\prime }\cdot 0\; & I=S\text{ and }n\neq 1,\\ w_{4}^{\prime }\cdot \mathrm{size}\left(B_{1},n\right)+w_{5}^{\prime }\cdot \mathrm{size}\left(B_{2},n\right)+w_{6}^{\prime }\cdot \mathrm{size}\left(B_{3},n\right)\; & I=B,\\ w_{7}^{\prime }\cdot \mathrm{size}\left(C_{1},n\right)+w_{8}^{\prime }\cdot \mathrm{size}\left(C_{2},n\right)\; & I=C,\\ 0\; & \text{else}. \end{array}\right)$$

This recursive size function (with weighted class sizes) can now be used for the straightforward construction of a corresponding algorithm for the non-uniform generation of elements of $L\left(G_{d}\right)$ by means of unranking, as proposed in [20].

### Derivation of the Algorithm

In this section, we give a complete and detailled description of the derivation of our weighted unranking algorithm for RNA secondary structures. The different steps are made according to the approach described in [20] to get an unranking algorithm that generates random RNA secondary structures of a given size *n *according to the distribution on all these structures.

#### Considered (unambiguous, *ϵ*-free and loop-free) SCFG

First, note that in [21], to obtain the stochastic model for RNA secondary structures derived from real-world RNA data, the following unambiguous SCFG which unambiguously generates exactly the language  given in Definition 0.9 has been used:

**Definition App-.11**. The unambiguous SCFG $G_{\text{sto}}$ generating exactly the language  is given by $G_{\text{sto}}=\left(I_{G_{\text{sto}}},\;\sum_{G_{\text{sto}}},\;R_{G_{\text{sto}}},\;S\right)$, where

$$I_{G_{sto}}=\left\{S,T,C,A,L,G,B,F,H,P,Q,R,J,K,M,N,U\right\},$$

$\Sigma_{G_{\text{sto}}}=\left\{\left(,\right),\;|\right\}$ and $R_{G_{\text{sto}}}$ contains exactly the following rules:

$$\begin{array}{cccccccc} p_{1}: & S\to TAC,\\ p_{2}: & T\to TAC, & p_{3}: & T\to C,\\ p_{4}: & C\to C|, & p_{5}: & C\to \epsilon ,\\ p_{6}: & A\to \left(L\right),\\ p_{7}: & L\to \left(L\right), & p_{8}: & L\to M, & p_{9}: & L\to P, & p_{10}: & L\to Q,\\ p_{11}: & L\to R, & p_{12}: & L\to F, & p_{13}: & L\to G,\\ p_{14}: & G\to \left(L\right)|, & p_{15}: & G\to \left(L\right)B||, & p_{16}: & G\to |\left(L\right), & p_{17}: & G\to ||B\left(L\right),\\ p_{18}: & B\to B|, & p_{19}: & B\to \epsilon ,\\ p_{20}: & F\to |||, & p_{21}: & F\to ||||, & p_{22}: & F\to |||||H,\\ p_{23}: & H\to H|, & p_{24}: & H\to \epsilon ,\\ p_{25}: & P\to |\left(L\right)|, & p_{26}: & P\to |\left(L\right)||, & p_{27}: & P\to ||\left(L\right)|, & p_{28}: & P\to ||\left(L\right)||,\\ p_{29}: & Q\to ||\left(L\right)K|||, & p_{30}: & Q\to |||J\left(L\right)K||,\\ p_{31}: & R\to |\left(L\right)K|||, & p_{32}: & R\to |||J\left(L\right)|,\\ p_{33}: & J\to J|, & p_{34}: & J\to \epsilon ,\\ p_{35}: & K\to K|, & p_{36}: & K\to \epsilon ,\\ p_{37}: & M\to U\left(L\right)U\left(L\right)N,\\ p_{38}: & N\to U\left(L\right)N, & p_{39}: & N\to U,\\ p_{40}: & U\to U|, & p_{41}: & U\to \epsilon . \end{array}$$

In this grammar, different intermediate symbols have been used to distinguish between different substructures. In fact, the reason why this grammar has so many production rules is that the grammar must be able to distinguish between all the different classes of substructures for which there are different free energy rules according to Turner's thermodynamic model considered in [21].

However, as *ϵ*-freeness and loop-freeness are required preliminarily, we have to consider another unambiguous SCFG generating the same language , where we have to guarantee that the same substructures are distinguished as are distinguished in $G_{\text{sto}}$.

Using the usual way of transforming a non-*ϵ*-free grammar into an *ϵ*-free one, the following definition can immediately be obtained from the previous one:

**Definition App-.12**. The unambigous and *ϵ*-free SCFG $G_{\text{sto}}^{\prime }$ generating exactly the language  is given by $G_{sto}^{\prime }=(I_{G_{sto}^{\prime }},\Sigma_{G_{sto}^{\prime }},R_{G_{sto}^{\prime }},S^{\prime })$, where

$$I_{G_{sto}^{\prime }}=\left\{S\prime ,S,T,C,A,L,G,B,F,H,P,Q,R,J,K,M,N,U\right\},$$

$\Sigma_{G_{\text{sto}}^{\prime }}=\left\{\left(,\right),\;|\right\}$ and $R_{G_{sto}^{\prime }}$ contains exactly the following rules:

$$\begin{array}{cccccccc} p_{0}^{\prime }: & S^{\prime }\to S,\\ p_{1}^{\prime }: & S\to A, & p_{2}^{\prime }: & S\to AC, & p_{3}^{\prime }: & S\to TA, & p_{4}^{\prime }: & S\to TAC,\\ p_{5}^{\prime }: & T\to A, & p_{6}^{\prime }: & T\to AC, & p_{7}^{\prime }: & T\to TA, & p_{8}^{\prime }: & T\to TAC,\\ p_{9}^{\prime }: & T\to C,\\ p_{10}^{\prime }: & C\to |, & p_{11}^{\prime }: & C\to C|,\\ p_{12}^{\prime }: & A\to \left(L\right),\\ p_{13}^{\prime }: & L\to \left(L\right), & p_{14}^{\prime }: & L\to M, & p_{15}^{\prime }: & L\to P, & p_{16}^{\prime }: & L\to Q,\\ p_{17}^{\prime }: & L\to R, & p_{18}^{\prime }: & L\to F, & p_{19}^{\prime }: & L\to G,\\ p_{20}^{\prime }: & G\to \left(L\right)|, & p_{21}^{\prime }: & G\to \left(L\right)||, & p_{22}^{\prime }: & G\to \left(L\right)B||,\\ p_{23}^{\prime }: & G\to |\left(L\right), & p_{24}^{\prime }: & G\to ||\left(L\right), & p_{25}^{\prime }: & G\to ||B\left(L\right),\\ p_{26}^{\prime }: & B\to |, & p_{27}^{\prime }: & B\to B|,\\ p_{28}^{\prime }: & F\to |||, & p_{29}^{\prime }: & F\to ||||, & p_{30}^{\prime }: & F\to |||||, & p_{31}^{\prime }: & F\to |||||H,\\ p_{32}^{\prime }: & H\to |, & p_{33}^{\prime }: & H\to H|,\\ p_{34}^{\prime }: & P\to |\left(L\right)|, & p_{35}^{\prime }: & P\to |\left(L\right)||, & p_{36}^{\prime }: & P\to ||\left(L\right)|, & p_{37}^{\prime }: & P\to ||\left(L\right)||,\\ p_{38}^{\prime }: & Q\to ||\left(L\right)|||, & p_{39}^{\prime }: & Q\to ||\left(L\right)K|||, & p_{40}^{\prime }: & Q\to |||\left(L\right)||, & p_{41}^{\prime }: & Q\to |||J\left(L\right)||,\\ p_{42}^{\prime }: & Q\to |||\left(L\right)K||, & p_{43}^{\prime }: & Q\to |||J\left(L\right)K||,\\ p_{44}^{\prime }: & R\to |\left(L\right)|||, & p_{45}^{\prime }: & R\to |\left(L\right)K|||, & p_{46}^{\prime }: & R\to |||\left(L\right)|, & p_{47}^{\prime }: & R\to |||J\left(L\right)|,\\ p_{48}^{\prime }: & J\to |, & p_{49}^{\prime }: & J\to J|,\\ p_{50}^{\prime }: & K\to |, & p_{51}^{\prime }: & K\to K|,\\ p_{52}^{\prime }: & M\to \left(L\right)\left(L\right), & p_{53}^{\prime }: & M\to U\left(L\right)\left(L\right), & p_{54}^{\prime }: & M\to \left(L\right)U\left(L\right), & p_{55}^{\prime }: & M\to \left(L\right)\left(L\right)N,\\ p_{56}^{\prime }: & M\to U\left(L\right)U\left(L\right), & p_{57}^{\prime }: & M\to U\left(L\right)\left(L\right)N, & p_{58}^{\prime }: & M\to \left(L\right)U\left(L\right)N, & p_{59}^{\prime }: & M\to U\left(L\right)U\left(L\right)N,\\ p_{60}^{\prime }: & N\to \left(L\right), & p_{61}^{\prime }: & N\to U\left(L\right), & p_{62}^{\prime }: & N\to \left(L\right)N, & p_{63}^{\prime }: & N\to U\left(L\right)N,\\ p_{64}^{\prime }: & N\to U,\\ p_{65}^{\prime }: & U\to |, & p_{66}^{\prime }: & U\to U|. \end{array}$$

Unfortunately, the set of productions of $G_{\text{sto}}^{\prime }$ contains productions with up to 5 non-terminal symbols in the conclusion. This is not acceptable for our purpose, for the following reason: the desired unranking algorithm makes use of the size of combinatorial classes whose representations somehow are derived from CFGs with particular integer weights on their productions. If we constructed this WCFG by starting with the grammar $G_{\text{sto}}^{\prime }$, then this would yield a huge number of production rules. Consequently, the translation would imply a huge specification of the combinatorial classes and the corresponding function to compute their sizes and thus the corresponding unranking algorithm would have to distinguish between an unnecessarily and most importantly unacceptably large number of cases.

Nevertheless, the size of the production set of the weighted grammar underlying the desired unranking algorithm can be significantly reduced by starting with a modification of grammar $G_{\text{sto}}^{\prime }$ which has only production rules with minimum possible numbers of non-terminal symbols in the conclusion. In fact, by transforming $G_{\text{sto}}^{\prime }$ appropriately considering this observation, we obtained the SCFG ${Ĝ}_{\text{sto}}$:

**Definition App-.13**. The unambiguous *ϵ*-free SCFG ${\hat{G}}_{sto}$ generating exactly the language  is given by ${\hat{G}}_{sto}=(I_{{\hat{G}}_{sto}},\Sigma_{{\hat{G}}_{sto}},R_{{\hat{G}}_{sto}},S^{\prime })$, where

$$I_{{\hat{G}}_{\text{sto}}}=\left\{S\prime ,E,S,T,C,A,L,G,D,B,F,H,P,Q,R,V,W,O,J,K,M,X,Y,Z,N,U\right\},$$

$\sum_{{\hat{G}}_{\text{sto}}}=\left\{\left(,\right),|\right)$ and $R_{{\hat{G}}_{\text{sto}}}$ contains exactly the following rules:

$$\begin{array}{cccccccc} {\hat{p}}_{1}: & S^{\prime }\to E,\\ {\hat{p}}_{2}: & E\to S, & {\hat{p}}_{3}: & E\to SC,\\ {\hat{p}}_{4}: & S\to A, & {\hat{p}}_{5}: & S\to TA,\\ {\hat{p}}_{6}: & T\to E, & {\hat{p}}_{7}: & T\to C,\\ {\hat{p}}_{8}: & C\to |, & {\hat{p}}_{9}: & C\to C|,\\ {\hat{p}}_{10}: & A\to \left(L\right),\\ {\hat{p}}_{11}: & L\to A, & {\hat{p}}_{12}: & L\to M, & {\hat{p}}_{13}: & L\to P, & {\hat{p}}_{14}: & L\to Q,\\ {\hat{p}}_{15}: & L\to R, & {\hat{p}}_{16}: & L\to F, & {\hat{p}}_{17}: & L\to G,\\ {\hat{p}}_{18}: & G\to A|, & {\hat{p}}_{19}: & G\to AD, & {\hat{p}}_{20}: & G\to |A, & {\hat{p}}_{21}: & G\to DA,\\ {\hat{p}}_{22}: & D\to B|,\\ {\hat{p}}_{23}: & B\to |, & {\hat{p}}_{24}: & B\to B|,\\ {\hat{p}}_{25}: & F\to |||, & {\hat{p}}_{26}: & F\to ||||, & {\hat{p}}_{27}: & F\to ||||H,\\ {\hat{p}}_{28}: & H\to |, & {\hat{p}}_{29}: & H\to H|,\\ {\hat{p}}_{30}: & P\to |A|, & {\hat{p}}_{31}: & P\to |A||, & {\hat{p}}_{32}: & P\to ||A|, & {\hat{p}}_{33}: & P\to ||A||,\\ {\hat{p}}_{34}: & Q\to ||O||, & {\hat{p}}_{35}: & Q\to ||V|,\\ {\hat{p}}_{36}: & R\to |O||, & {\hat{p}}_{37}: & R\to ||W|,\\ {\hat{p}}_{38}: & V\to JO,\\ {\hat{p}}_{39}: & W\to JA,\\ {\hat{p}}_{40}: & O\to AK,\\ {\hat{p}}_{41}: & J\to |, & {\hat{p}}_{42}: & J\to J|,\\ {\hat{p}}_{43}: & K\to |, & {\hat{p}}_{44}: & K\to K|,\\ {\hat{p}}_{45}: & M\to XY,\\ {\hat{p}}_{46}: & X\to A, & {\hat{p}}_{47}: & X\to UA,\\ {\hat{p}}_{48}: & Y\to Z,\\ {\hat{p}}_{49}: & Z\to X, & {\hat{p}}_{50}: & Z\to XN,\\ {\hat{p}}_{51}: & N\to Z, & {\hat{p}}_{52}: & N\to U,\\ {\hat{p}}_{53}: & U\to |, & {\hat{p}}_{54}: & U\to U|.\\ : \end{array}$$

#### Transforming our SCFG into RNF

Now, we can construct the desired weighted grammar that will be underlying our unranking algorithm: In the first step, we gather all possible chains of productions that do not lengthen the sentential form. In fact, we have to consider all rules *A *→ *α*, *A *≠ *S'*, with |*α*| = 1, to obtain all such chains (note that these rules will be removed after step 1). Hence, we have to consider the following set $R_{rnf}^{1}$ of 22 production rules:

$$\begin{array}{cccccccc} {\hat{p}}_{2}: & E\to S,\\ {\hat{p}}_{4}: & S\to A,\\ {\hat{p}}_{6}: & T\to E, & {\hat{p}}_{7}: & T\to C,\\ {\hat{p}}_{8}: & C\to |,\\ {\hat{p}}_{11}: & L\to A, & {\hat{p}}_{12}: & L\to M, & {\hat{p}}_{13}: & L\to P, & {\hat{p}}_{14}: & L\to Q,\\ {\hat{p}}_{15}: & L\to R, & {\hat{p}}_{16}: & L\to F, & {\hat{p}}_{17}: & L\to G,\\ {\hat{p}}_{23}: & B\to |,\\ {\hat{p}}_{28}: & H\to |,\\ {\hat{p}}_{41}: & J\to |,\\ {\hat{p}}_{43}: & K\to |,\\ {\hat{p}}_{46}: & X\to A,\\ {\hat{p}}_{48}: & Y\to Z,\\ {\hat{p}}_{49}: & Z\to X,\\ {\hat{p}}_{51}: & N\to Z, & {\hat{p}}_{52}: & N\to U,\\ {\hat{p}}_{53}: & U\to |. \end{array}$$

Thus, the following 32 chains are gathered in step 1:

$$\begin{array}{cc} E\Rightarrow S, & targets\left[E\right]=\{\left(S,\lambda_{E,S}:={\hat{p}}_{2},\epsilon \right),\\ E\Rightarrow S\Rightarrow A, & \;\;\;\;\;\;\;\;\;\;\;\;\;\;\;\;\;\;\;\left(A,\lambda_{E,A}:={\hat{p}}_{2}\cdot {\hat{p}}_{4},S\right)\},\\ S\Rightarrow A, & targets\left[S\right]=\left\{\left(A,\lambda_{S,A}:={\hat{p}}_{4},\epsilon \right)\right\},\\ T\Rightarrow E, & targets\left[T\right]=\{\left(E,\lambda_{T,E}:={\hat{p}}_{6},\epsilon \right),\\ T\Rightarrow C, & \;\;\;\;\;\;\;\;\;\;\;\;\;\;\;\;\;\;\;\left(C,\lambda_{T,C}:={\hat{p}}_{7},\epsilon \right),\\ T\Rightarrow C\Rightarrow |, & \;\;\;\;\;\;\;\;\;\;\;\;\;\;\;\;\;\;\;\left(|,\lambda_{T,|}:={\hat{p}}_{7}\cdot {\hat{p}}_{8},C\right),\\ T\Rightarrow E\Rightarrow S, & \;\;\;\;\;\;\;\;\;\;\;\;\;\;\;\;\;\;\;\left(S,\lambda_{T,S}:={\hat{p}}_{6}\cdot {\hat{p}}_{2},E\right)\},\\ T\Rightarrow E\Rightarrow S\Rightarrow A, & \;\;\;\;\;\;\;\;\;\;\;\;\;\;\;\;\;\;\;(A,\lambda_{T,A}:={\hat{p}}_{6}\cdot {\hat{p}}_{2}\cdot {\hat{p}}_{4},ES)\},\\ C\Rightarrow |, & targets\left[C\right]=\left\{\left(|,\lambda_{C,|}:={\hat{p}}_{8},\epsilon \right)\right\},\\ L\Rightarrow A, & targets\left[L\right]=\{\left(A,\lambda_{L,A}:={\hat{p}}_{11},\epsilon \right),\\ L\Rightarrow M, & \;\;\;\;\;\;\;\;\;\;\;\;\;\;\;\;\;\;\;\left(M,\lambda_{L,M}:={\hat{p}}_{12},\epsilon \right),\\ L\Rightarrow P, & \;\;\;\;\;\;\;\;\;\;\;\;\;\;\;\;\;\;\;\left(P,\lambda_{L,P}:={\hat{p}}_{13},\epsilon \right),\\ L\Rightarrow Q, & \;\;\;\;\;\;\;\;\;\;\;\;\;\;\;\;\;\;\;\left(Q,\lambda_{L,Q}:={\hat{p}}_{14},\epsilon \right),\\ L\Rightarrow R, & \;\;\;\;\;\;\;\;\;\;\;\;\;\;\;\;\;\;\;\left(R,\lambda_{L,R}:={\hat{p}}_{15},\epsilon \right),\\ L\Rightarrow F, & \;\;\;\;\;\;\;\;\;\;\;\;\;\;\;\;\;\;\;\left(F,\lambda_{L,F}:={\hat{p}}_{16},\epsilon \right),\\ L\Rightarrow G, & \;\;\;\;\;\;\;\;\;\;\;\;\;\;\;\;\;\;\;\left(G,\lambda_{L,G}:={\hat{p}}_{17},\epsilon \right)\},\\ B\Rightarrow |, & targets\left[B\right]=\left\{\left(|,\lambda_{B,|}:={\hat{p}}_{23},\epsilon \right)\right\},\\ H\Rightarrow |, & targets\left[H\right]=\left\{\left(|,\lambda_{H,|}:={\hat{p}}_{28},\epsilon \right)\right\},\\ J\Rightarrow |, & targets\left[J\right]=\left\{\left(|,\lambda_{J,|}:={\hat{p}}_{41},\epsilon \right)\right\},\\ K\Rightarrow |, & targets\left[K\right]=\left\{\left(|,\lambda_{K,|}:={\hat{p}}_{43},\epsilon \right)\right\},\\ X\Rightarrow A, & targets\left[X\right]=\left\{\left(A,\lambda_{X,A}:={\hat{p}}_{46},\epsilon \right)\right\},\\ Y\Rightarrow Z, & targets\left[Y\right]=\{\left(Z,\lambda_{Y,Z}:={\hat{p}}_{48},\epsilon \right),\\ Y\Rightarrow Z\Rightarrow X, & \;\;\;\;\;\;\;\;\;\;\;\;\;\;\;\;\;\;\;\left(X,\lambda_{Y,X}:={\hat{p}}_{48}\cdot {\hat{p}}_{49},Z\right),\\ Y\Rightarrow Z\Rightarrow X\Rightarrow A, & \;\;\;\;\;\;\;\;\;\;\;\;\;\;\;\;\;\;\;\left(A,\lambda_{Y,A}:={\hat{p}}_{48}\cdot {\hat{p}}_{49}\cdot {\hat{p}}_{46},ZX\right)\},\\ Z\Rightarrow X, & targets\left[Z\right]=\{\left(X,\lambda_{Z,X}:={\hat{p}}_{49},\epsilon \right),\\ Z\Rightarrow X\Rightarrow A, & \;\;\;\;\;\;\;\;\;\;\;\;\;\;\;\;\;\;\;(A,\lambda_{Z,A}:={\hat{p}}_{49}\cdot {\hat{p}}_{46},X)\},\\ N\Rightarrow Z, & targets\left[N\right]=\{\left(Z,\lambda_{N,Z}:={\hat{p}}_{51},\epsilon \right),\\ N\Rightarrow U, & \;\;\;\;\;\;\;\;\;\;\;\;\;\;\;\;\;\;\;\left(U,\lambda_{N,U}:={\hat{p}}_{52},\epsilon \right),\\ N\Rightarrow U\Rightarrow |, & \;\;\;\;\;\;\;\;\;\;\;\;\;\;\;\;\;\;\;\left(|,\lambda_{N,|}:={\hat{p}}_{52}\cdot {\hat{p}}_{53},U\right),\\ N\Rightarrow Z\Rightarrow X, & \;\;\;\;\;\;\;\;\;\;\;\;\;\;\;\;\;\;\;\left(X,\lambda_{N,X}:={\hat{p}}_{51}\cdot {\hat{p}}_{49},Z\right),\\ N\Rightarrow Z\Rightarrow X\Rightarrow A, & \;\;\;\;\;\;\;\;\;\;\;\;\;\;\;\;\;\;\;(A,\lambda_{N,A}:={\hat{p}}_{51}\cdot {\hat{p}}_{49}\cdot {\hat{p}}_{46},ZX)\},\\ U\Rightarrow |, & targets\left[U\right]=\left\{\left(|,\lambda_{U,|}:={\hat{p}}_{53},\epsilon \right)\right\}. \end{array}$$

Furthermore, the 22 production rules contained in $R_{rnf}^{1}$ are now removed. This results in the following set $R_{{Ĝ}_{\text{sto}}}^{1}:=R_{{Ĝ}_{\text{sto}}}\backslash R_{rnf}^{1}$ of 32 rules:

$$\begin{array}{cccccccc} {\hat{p}}_{1}: & S^{\prime }\to E,\\ {\hat{p}}_{3}: & E\to SC,\\ {\hat{p}}_{5}: & S\to TA,\\ {\hat{p}}_{9}: & C\to C|,\\ {\hat{p}}_{10}: & A\to \left(L\right),\\ {\hat{p}}_{18}: & G\to A|, & {\hat{p}}_{19}: & G\to AD, & {\hat{p}}_{20}: & G\to |A, & {\hat{p}}_{21}: & G\to DA,\\ {\hat{p}}_{22}: & D\to B|,\\ {\hat{p}}_{24}: & B\to B|,\\ {\hat{p}}_{25}: & F\to |||, & {\hat{p}}_{26}: & F\to ||||, & {\hat{p}}_{27}: & F\to ||||H,\\ {\hat{p}}_{29}: & H\to H|,\\ {\hat{p}}_{30}: & P\to |A|, & {\hat{p}}_{31}: & P\to |A||, & {\hat{p}}_{32}: & P\to ||A|, & {\hat{p}}_{33}: & P\to ||A||,\\ {\hat{p}}_{34}: & Q\to ||O||, & {\hat{p}}_{35}: & Q\to ||V|,\\ {\hat{p}}_{36}: & R\to |O||, & {\hat{p}}_{37}: & R\to ||W|,\\ {\hat{p}}_{38}: & V\to JO,\\ {\hat{p}}_{39}: & W\to JA,\\ {\hat{p}}_{40}: & O\to AK,\\ {\hat{p}}_{42}: & J\to J|,\\ {\hat{p}}_{44}: & K\to K|,\\ {\hat{p}}_{45}: & M\to XY,\\ {\hat{p}}_{47}: & X\to UA,\\ {\hat{p}}_{50}: & Z\to XN,\\ {\hat{p}}_{54}: & U\to U|. \end{array}$$

Additionally, in step 2, for each chain a new intermediate symbol and a new production are introduced. Thus, according to the 32 chains gathered in step 1, we here obtain the following set $R_{rnf}^{2}$ of 32 new production rules:

$$\begin{array}{cccccccc} 1: & E^{S,\epsilon }\to S, & 1: & E^{A,S}\to A,\\ 1: & S^{A,\epsilon }\to A,\\ 1: & T^{E,\epsilon }\to E, & 1: & T^{C,\epsilon }\to C, & 1: & T^{|,C}\to |,\\ 1: & T^{S,E}\to S, & 1: & T^{A,ES}\to A,\\ 1: & C^{|,\epsilon }\to |,\\ 1: & L^{A,\epsilon }\to A, & 1: & L^{M,\epsilon }\to M, & 1: & L^{P,\epsilon }\to P, & 1: & L^{Q,\epsilon }\to Q,\\ 1: & L^{R,\epsilon }\to R, & 1: & L^{F,\epsilon }\to F, & 1: & L^{G,\epsilon }\to G,\\ 1: & B^{|,\epsilon }\to |,\\ 1: & H^{|,\epsilon }\to |,\\ 1: & J^{|,\epsilon }\to |,\\ 1: & K^{|,\epsilon }\to |,\\ 1: & X^{A,\epsilon }\to A,\\ 1: & Y^{Z,\epsilon }\to Z, & 1: & Y^{X,Z}\to X, & 1: & Y^{A,ZX}\to A,\\ 1: & Z^{X,\epsilon }\to X,\\ 1: & Z^{A,X}\to A,\\ 1: & N^{Z,\epsilon }\to Z, & 1: & N^{U,\epsilon }\to U, & 1: & N^{|,U}\to |,\\ 1: & N^{X,Z}\to X, & 1: & N^{A,ZX}\to A,\\ 1: & U^{|,\epsilon }\to |. \end{array}$$

In step 3, for each occurrence of a non-terminal symbol in the conclusion of a production and each chain starting with this non-terminal symbol, we have to add a new production with the corresponding new intermediate symbol instead of the considered one. Thus, in step 3, the remaining 32 production rules from $R_{{Ĝ}_{\text{sto}}}^{1}:=R_{{Ĝ}_{\text{sto}}}\backslash R_{rnf}^{1}$ are transformed (according to $R_{rnf}^{2}$) into the following set $R_{{Ĝ}_{\text{sto}}}^{2}$ of 79 new rules:

$$\begin{array}{cccc} {\hat{p}}_{1}: & S^{\prime }\to E, & {\hat{p}}_{1}\cdot \lambda_{E,S}: & S^{\prime }\to E^{S,\epsilon },\\ {\hat{p}}_{1}\cdot \lambda_{E,A}: & S^{\prime }\to E^{A,S},\\ {\hat{p}}_{3}: & E\to SC, & {\hat{p}}_{3}\cdot \lambda_{S,A}: & E\to S^{A,\epsilon }C,\\ {\hat{p}}_{3}\cdot \lambda_{C,|}: & E\to SC^{|,\epsilon }, & {\hat{p}}_{3}\cdot \lambda_{S,A}\cdot \lambda_{C,|}: & E\to S^{A,\epsilon }C^{|,\epsilon },\\ {\hat{p}}_{5}: & S\to TA, & {\hat{p}}_{5}\cdot \lambda_{T,E}: & S\to T^{E,\epsilon }A,\\ {\hat{p}}_{5}\cdot \lambda_{T,C}: & S\to T^{C,\epsilon }A, & {\hat{p}}_{5}\cdot \lambda_{T,|}: & S\to T^{|,C}A,\\ {\hat{p}}_{5}\cdot \lambda_{T,S}: & S\to T^{S,E}A, & {\hat{p}}_{5}\cdot \lambda_{T,A}: & S\to T^{A,ES}A,\\ {\hat{p}}_{9}: & C\to C|, & {\hat{p}}_{9}\cdot \lambda_{C,|}: & C\to C^{|,\epsilon }|,\\ {\hat{p}}_{10}: & A\to \left(L\right), & {\hat{p}}_{10}\cdot \lambda_{L,A}: & A\to \left(L^{A,\epsilon }\right),\\ {\hat{p}}_{10}\cdot \lambda_{L,M}: & A\to \left(L^{M,\epsilon }\right), & {\hat{p}}_{10}\cdot \lambda_{L,P}: & A\to \left(L^{P,\epsilon }\right),\\ {\hat{p}}_{10}\cdot \lambda_{L,Q}: & A\to \left(L^{Q,\epsilon }\right), & {\hat{p}}_{10}\cdot \lambda_{L,R}: & A\to \left(L^{R,\epsilon }\right),\\ {\hat{p}}_{10}\cdot \lambda_{L,F}: & A\to \left(L^{F,\epsilon }\right), & {\hat{p}}_{10}\cdot \lambda_{L,G}: & A\to \left(L^{G,\epsilon }\right),\\ {\hat{p}}_{18}: & G\to A|, & {\hat{p}}_{19}: & G\to AD,\\ {\hat{p}}_{20}: & G\to |A, & {\hat{p}}_{21}: & G\to DA,\\ {\hat{p}}_{22}: & D\to B|, & {\hat{p}}_{22}\cdot \lambda_{B,|}: & D\to B^{|,\epsilon }|,\\ {\hat{p}}_{24}: & B\to B|, & {\hat{p}}_{24}\cdot \lambda_{B,|}: & B\to B^{|,\epsilon }|,\\ {\hat{p}}_{25}: & F\to |||, & {\hat{p}}_{26}: & F\to ||||,\\ {\hat{p}}_{27}: & F\to ||||H, & {\hat{p}}_{27}\cdot \lambda_{H,|}: & F\to ||||H^{|,\epsilon },\\ {\hat{p}}_{29}: & H\to H|, & {\hat{p}}_{29}\cdot \lambda_{H,|}: & H\to H^{|,\epsilon }|,\\ {\hat{p}}_{30}: & P\to |A|, & {\hat{p}}_{31}: & P\to |A||,\\ {\hat{p}}_{32}: & P\to ||A|, & {\hat{p}}_{33}: & P\to ||A||,\\ {\hat{p}}_{34}: & Q\to ||O||, & {\hat{p}}_{35}: & Q\to ||V|,\\ {\hat{p}}_{36}: & R\to |O||, & {\hat{p}}_{37}: & R\to ||W|,\\ {\hat{p}}_{38}: & V\to JO, & {\hat{p}}_{38}\cdot \lambda_{J,|}: & V\to J^{|,\epsilon }O,\\ {\hat{p}}_{39}: & W\to JA, & {\hat{p}}_{39}\cdot \lambda_{J,|}: & W\to J^{|,\epsilon }A,\\ {\hat{p}}_{40}: & O\to AK, & {\hat{p}}_{40}\cdot \lambda_{K,|}: & O\to AK^{|,\epsilon },\\ {\hat{p}}_{42}: & J\to J|, & {\hat{p}}_{42}\cdot \lambda_{J,|}: & J\to J^{|,\epsilon }|,\\ {\hat{p}}_{44}: & K\to K|, & {\hat{p}}_{44}\cdot \lambda_{K,|}: & K\to K^{|,\epsilon }|,\\ {\hat{p}}_{45}: & M\to XY, & {\hat{p}}_{45}\cdot \lambda_{Y,Z}: & M\to XY^{Z,\epsilon },\\ {\hat{p}}_{45}\cdot \lambda_{Y,X}: & M\to XY^{X,Z}, & {\hat{p}}_{45}\cdot \lambda_{Y,A}: & M\to XY^{A,ZX},\\ {\hat{p}}_{45}\cdot \lambda_{X,A}: & M\to X^{A,\epsilon }Y, & {\hat{p}}_{45}\cdot \lambda_{X,A}\cdot \lambda_{Y,Z}: & M\to X^{A,\epsilon }Y^{Z,\epsilon },\\ {\hat{p}}_{45}\cdot \lambda_{X,A}\cdot \lambda_{Y,X}: & M\to X^{A,\epsilon }Y^{X,Z}, & {\hat{p}}_{45}\cdot \lambda_{X,A}\cdot \lambda_{Y,A}: & M\to X^{A,\epsilon }Y^{A,ZX},\\ {\hat{p}}_{47}: & X\to UA, & {\hat{p}}_{47}\cdot \lambda_{U,|}: & X\to U^{|,\epsilon }A,\\ {\hat{p}}_{50}: & Z\to XN, & {\hat{p}}_{50}\cdot \lambda_{N,Z}: & Z\to XN^{Z,\epsilon },\\ {\hat{p}}_{50}\cdot \lambda_{N,U}: & Z\to XN^{U,\epsilon }, & {\hat{p}}_{50}\cdot \lambda_{N,|}: & Z\to XN^{|,U},\\ {\hat{p}}_{50}\cdot \lambda_{N,X}: & Z\to XN^{X,Z}, & {\hat{p}}_{50}\cdot \lambda_{N,A}: & Z\to XN^{A,ZX},\\ {\hat{p}}_{50}\cdot \lambda_{X,A}: & Z\to X^{A,\epsilon }N, & {\hat{p}}_{50}\cdot \lambda_{X,A}\cdot \lambda_{N,Z}: & Z\to X^{A,\epsilon }N^{Z,\epsilon },\\ {\hat{p}}_{50}\cdot \lambda_{X,A}\cdot \lambda_{N,U}: & Z\to X^{A,\epsilon }N^{U,\epsilon }, & {\hat{p}}_{50}\cdot \lambda_{X,A}\cdot \lambda_{N,|}: & Z\to X^{A,\epsilon }N^{|,U},\\ {\hat{p}}_{50}\cdot \lambda_{X,A}\cdot \lambda_{N,X}: & Z\to X^{A,\epsilon }N^{X,Z}, & {\hat{p}}_{50}\cdot \lambda_{X,A}\cdot \lambda_{N,A}: & Z\to X^{A,\epsilon }N^{A,ZX},\\ {\hat{p}}_{54}: & U\to U|, & {\hat{p}}_{54}\cdot \lambda_{U,|}: & U\to U^{|,\epsilon }|. \end{array}$$

In step 4, we must delete all intermediate symbols that no longer occur as premise. Obviously, intermediate symbols no longer occurring as premise of a production are

T,L,N,Y.

We easily observe that the productions that contain at least one of these 4 intermediate symbols in the conclusion and thus have to be removed are exactly the following ones:

$$\begin{array}{cccc} {\hat{p}}_{5}: & S\to TA,\\ {\hat{p}}_{10}: & A\to \left(L\right),\\ {\hat{p}}_{45}: & M\to XY, & {\hat{p}}_{45}\cdot \lambda_{X,A}: & M\to X^{A,\epsilon }Y,\\ {\hat{p}}_{50}: & Z\to XN, & {\hat{p}}_{50}\cdot \lambda_{X,A}: & Z\to X^{A,\epsilon }N. \end{array}$$

Consequently, after the removal of these 6 rules from $R_{{Ĝ}_{\text{sto}}}^{2}$, there still remain 73 new production rules.

Finally in step 5, we must make sure that the conclusion of all productions with premise *S' *(axiom of ${Ĝ}_{\text{sto}}$ that we started with) does not have a length greater than 1. However, since there is only one production with premise *S' *in our start grammar ${Ĝ}_{\text{sto}}$ and the conclusion of this production has size 1, there is nothing to do. Thus, the resulting new grammar is given by:

**Definition App-.14**. The WCFG ${\hat{G}}_{\text{sto}}^{*}$ generating exactly the language  is given by ${\hat{G}}_{\text{sto}}^{*}=\left(I_{{\hat{G}}_{\text{sto}}^{*}}\cup {I^{\prime }}_{{\hat{G}}_{\text{sto}}^{*}},\sum_{{\hat{G}}_{\text{sto}}^{*}},R_{{\hat{G}}_{\text{sto}}^{*}}\cup {R^{\prime }}_{{\hat{G}}_{\text{sto}}^{*}},S^{\prime }\right)$, where

$$I_{{\hat{G}}_{\text{sto}}^{*}}=\left\{S^{\prime },E,S,C,A,G,D,B,F,H,P,Q,R,V,W,O,J,K,M,X,Z,U\right\},$$

$$\begin{array}{c} I_{{\hat{G}}_{\text{sto}}^{*}}^{\prime }=\{E^{S,\varepsilon },E^{A,S},S^{A,\varepsilon },z\\ \;\;T^{E,\varepsilon },T^{C,\varepsilon },T^{|,C},T^{S,E},T^{A,ES},C^{|,\varepsilon },\\ \;\;L^{A,\varepsilon },L^{M,\varepsilon },L^{P,\varepsilon },L^{Q,\varepsilon },L^{R,\varepsilon },L^{F,\varepsilon },L^{G,\varepsilon },\\ \;\;B^{|,\varepsilon },H^{|,\varepsilon },J^{|,\varepsilon },K^{|,\varepsilon },\\ \;\;X^{A,\varepsilon },Y^{Z,\varepsilon },Y^{X,Z},Y^{A,ZX},Z^{X,\varepsilon },Z^{A,X}\\ \;\;N^{Z,\varepsilon },N^{U,\varepsilon },N^{|,U},N^{X,Z},N^{A,ZX},U^{|,\varepsilon }\}, \end{array}$$

$\sum_{{\hat{G}}_{\text{sto}}^{*}}=\left\{\left(,\right),|\right\}$ and $R_{{\hat{G}}_{\text{sto}}^{*}}$ contains exactly the following rules:

$$\begin{array}{cccccccc} \lambda_{1}: & S^{\prime }\to E, & \lambda_{2}: & S^{\prime }\to E^{S,\epsilon }, & \lambda_{3}: & S^{\prime }\to E^{A,S},\\ \lambda_{4}: & E\to SC, & \lambda_{5}: & E\to S^{A,\epsilon }C, & \lambda_{6}: & E\to SC^{|,\epsilon }, & \lambda_{7}: & E\to S^{A,\epsilon }C^{|,\epsilon },\\ \lambda_{8}: & S\to T^{E,\epsilon }A, & \lambda_{9}: & S\to T^{C,\epsilon }A, & \lambda_{10}: & S\to T^{|,C}A,\\ \lambda_{11}: & S\to T^{S,E}A, & \lambda_{12}: & S\to T^{A,ES}A,\\ \lambda_{13}: & C\to C|, & \lambda_{14}: & C\to C^{|,\epsilon }|,\\ \lambda_{15}: & A\to \left(L^{A,\epsilon }\right), & \lambda_{16}: & A\to \left(L^{M,\epsilon }\right), & \lambda_{17}: & A\to \left(L^{P,\epsilon }\right), & \lambda_{18}: & A\to \left(L^{Q,\epsilon }\right),\\ \lambda_{19}: & A\to \left(L^{R,\epsilon }\right), & \lambda_{20}: & A\to \left(L^{F,\epsilon }\right), & \lambda_{21}: & A\to \left(L^{G,\epsilon }\right),\\ \lambda_{22}: & G\to A|, & \lambda_{23}: & G\to AD, & \lambda_{24}: & G\to |A, & \lambda_{25}: & G\to DA,\\ \lambda_{26}: & D\to B|, & \lambda_{27}: & D\to B^{|,\epsilon }|,\\ \lambda_{28}: & B\to B|, & \lambda_{29}: & B\to B^{|,\epsilon }|,\\ \lambda_{30}: & F\to |||, & \lambda_{31}: & F\to ||||, & \lambda_{32}: & F\to ||||H, & \lambda_{33}: & F\to ||||H^{|,\epsilon },\\ \lambda_{34}: & H\to H|, & \lambda_{35}: & H\to H^{|,\epsilon }|,\\ \lambda_{36}: & P\to |A|, & \lambda_{37}: & P\to |A||, & \lambda_{38}: & P\to ||A|, & \lambda_{39}: & P\to ||A||,\\ \lambda_{40}: & Q\to ||O||, & \lambda_{41}: & Q\to ||V|,\\ \lambda_{42}: & R\to |O||, & \lambda_{43}: & R\to ||W|,\\ \lambda_{44}: & V\to JO, & \lambda_{45}: & V\to J^{|,\epsilon }O,\\ \lambda_{46}: & W\to JA, & \lambda_{47}: & W\to J^{|,\epsilon }A,\\ \lambda_{48}: & O\to AK, & \lambda_{49}: & O\to AK^{|,\epsilon },\\ \lambda_{50}: & J\to J|, & \lambda_{51}: & J\to J^{|,\epsilon }|,\\ \lambda_{52}: & K\to K|, & \lambda_{53}: & K\to K^{|,\epsilon }|,\\ \lambda_{54}: & M\to XY^{Z,\epsilon }, & \lambda_{55}: & M\to XY^{X,Z}, & \lambda_{56}: & M\to XY^{A,ZX},\\ \lambda_{57}: & M\to X^{A,\epsilon }Y^{Z,\epsilon }, & \lambda_{58}: & M\to X^{A,\epsilon }Y^{X,Z}, & \lambda_{59}: & M\to X^{A,\epsilon }Y^{A,ZX},\\ \lambda_{60}: & X\to UA, & \lambda_{61}: & X\to U^{|,\epsilon }A,\\ \lambda_{62}: & Z\to XN^{Z,\epsilon }, & \lambda_{63}: & Z\to XN^{U,\epsilon }, & \lambda_{64}: & Z\to XN^{|,U},\\ \lambda_{65}: & Z\to XN^{X,Z}, & \lambda_{66}: & Z\to XN^{A,ZX},\\ \lambda_{67}: & Z\to X^{A,\epsilon }N^{Z,\epsilon }, & \lambda_{68}: & Z\to X^{A,\epsilon }N^{U,\epsilon }, & \lambda_{69}: & Z\to X^{A,\epsilon }N^{|,U},\\ \lambda_{70}: & Z\to X^{A,\epsilon }N^{X,Z}, & \lambda_{71}: & Z\to X^{A,\epsilon }N^{A,ZX},\\ \lambda_{72}: & U\to U|, & \lambda_{73}: & U\to U^{|,\epsilon }|, \end{array}$$

whereas $R_{{\hat{G}}_{sto}^{*}}^{\prime }$ contains exactly the following rules:

$$\begin{array}{cccccccc} \lambda_{74}: & E^{S,\epsilon }\to S, & \lambda_{75}: & E^{A,S}\to A,\\ \lambda_{76}: & S^{A,\epsilon }\to A,\\ \lambda_{77}: & T^{E,\epsilon }\to E, & \lambda_{78}: & T^{C,\epsilon }\to C, & \lambda_{79}: & T^{|,C}\to |,\\ \lambda_{80}: & T^{S,E}\to S, & \lambda_{81}: & T^{A,ES}\to A,\\ \lambda_{82}: & C^{|,\epsilon }\to |,\\ \lambda_{83}: & L^{A,\epsilon }\to A, & \lambda_{84}: & L^{M,\epsilon }\to M, & \lambda_{85}: & L^{P,\epsilon }\to P, & \lambda_{86}: & L^{Q,\epsilon }\to Q,\\ \lambda_{87}: & L^{R,\epsilon }\to R, & \lambda_{88}: & L^{F,\epsilon }\to F, & \lambda_{89}: & L^{G,\epsilon }\to G,\\ \lambda_{90}: & B^{|,\epsilon }\to |,\\ \lambda_{91}: & H^{|,\epsilon }\to |,\\ \lambda_{92}: & J^{|,\epsilon }\to |,\\ \lambda_{93}: & K^{|,\epsilon }\to |,\\ \lambda_{94}: & X^{A,\epsilon }\to A,\\ \lambda_{95}: & Y^{Z,\epsilon }\to Z, & \lambda_{96}: & Y^{X,Z}\to X, & \lambda_{97}: & Y^{A,ZX}\to A,\\ \lambda_{98}: & Z^{X,\epsilon }\to X, & \lambda_{99}: & Z^{A,X}\to A,\\ \lambda_{100}: & N^{Z,\epsilon }\to Z, & \lambda_{101}: & N^{U,\epsilon }\to U, & \lambda_{102}: & N^{|,U}\to |,\\ \lambda_{103}: & N^{X,Z}\to X, & \lambda_{104}: & N^{A,ZX}\to A,\\ \lambda_{105}: & U^{|,\epsilon }\to |. \end{array}$$

#### Reweighting the Production Rules

Now, the weights of the 73 production rules given in the subset of productions $R_{{Ĝ}_{\text{sto}}^{*}}$ have to be reweighted. In order to achieve this goal, we first have to compute the two common denominators *s *and *c*, where *s *is the common denominator of the weights of productions with premise *S' *(i.e., of productions number 1 to 3), and c is the common denominator of the weights of the remaining productions (i.e., of productions number 4 to 73) of $R_{{Ĝ}_{\text{sto}}^{*}}$. Using the rounded probabilities (weights) for the production rules of ${Ĝ}_{\text{sto}}^{*}$ as given in Table 5, we immediately find the smallest common denominators to be *s *= 10,000 and *c *= 10,000.

**Table 5:** Floating point approximations of the probabilities (weights) $\lambda_{i},\;1\leq i\leq 73$, for the production rules of the grammar ${Ĝ}_{\text{sto}}^{*}$ (rounded to four decimal places)

Nonterminal *Nt*		Weights of Rules with Premise *Nt*		
S'	λ_1 _: = 1.0000,	λ_2 _: = 0.0212,	λ_3 _: = 0.0003,	
E	λ_4 _: = 0.9788,	λ_5 _: = 0.0134,	λ_6 _: = 0.0944,	λ_7 _: = 0.0013,
S	λ_8 _: = 0.8559,	λ_9 _: = 0.1304,	λ_10 _: = 0.0126,	λ_11 _: = 0.0181,
	λ_12 _: = 0.0002,			
C	λ_13 _: = 0.9036,	λ_14 _: = 0.0871,		
A	λ_15 _: = 0.7630,	λ_16 _: = 0.0402,	λ_17 _: = 0.0186,	λ_18 _: = 0.0367,
	λ_19 _: = 0.0072,	λ_20 _: = 0.0858,	λ_21 _: = 0.0484,	
G	λ_22 _: = 0.3038,	λ_23 _: = 0.1884,	λ_24 _: = 0.3081,	λ_25 _: = 0.1996,
D	λ_26 _: = 1.0000,	λ_27 _: = 0.3896,		
B	λ_28 _: = 0.6104,	λ_29 _: = 0.2378,		
F	λ_30 _: = 0.0575,	λ_31 _: = 0.3409,	λ_32 _: = 0.6016,	λ33: = 0.1211,
H	λ_34 _: = 0.7987,	λ_35 _: = 0.1608,		
P	λ_36 _: = 0.1085,	λ_37 _: = 0.2144,	λ_38 _: = 0.2011,	λ_39 _: = 0.4760,
Q	λ_40 _: = 0.1713,	λ_41 _: = 0.8287,		
R	λ_42 _: = 0.4150,	λ_43 _: = 0.5850,		
V	λ_44 _: = 1.0000,	λ_45 _: = 0.3243,		
W	λ_46 _: = 1.0000,	λ_47 _: = 0.3243,		
O	λ_48 _: = 1.0000,	λ_49 _: = 0.2928,		
J	λ_50 _: = 0.6757,	λ_51 _: = 0.2191,		
K	λ_52 _: = 0.7072,	λ_53 _: = 0.2071,		
M	λ_54 _: = 1.0000,	λ_55 _: = 0.0510,	λ_56 _: = 0.0036,	λ57: = 0.0712,
	λ_58 _: = 0.0036,	λ_59 _: = 0.0003,		
X	λ_60 _: = 0.9288,	λ_61 _: = 0.1968,		
Z	λ_62 _: = 0.4211,	λ_63 _: = 0.5279,	λ_64 _: = 0.1119,	λ_65 _: = 0.0215,
	λ_66 _: = 0.0015,	λ_67 _: = 0.0300,	λ_68 _: = 0.0376,	λ_69 _: = 0.0080,
	λ_70 _: = 0.0015,	λ_71 _: = 0.0001,		
U	λ_72 _: = 0.7881,	λ_73 _: = 0.1670.		

Note that for $i\in \{74,...,105\},\;\lambda_{i}:=1$ holds.

The desired new weights for the considered set of productions $R_{{Ĝ}_{\text{sto}}^{*}}$ are then computed by multiplying the old weights of productions with source *S' *by *s*, and by multiplying the old weights of productions *A *→ *α*, *A *≠ *S' *(and $A\in I_{{Ĝ}_{\text{sto}}^{*}}$), by *c*^|α|-1^.

Formally, for the reweighted set of productions $R_{{Ĝ}_{\text{sto}}^{*}}$, we get the following weights:

$$\mu_{i}:=\lambda_{i}\cdot s,\;\text{for}\;i\in \left\{1,2,3\right\}$$

and

$$\mu_{i}:=\lambda_{i}\cdot c^{|\alpha_{i}|-1},\;\;\text{where}\;\;\lambda_{i}:A_{i}\to \alpha_{i},\;\text{for}\;i\in \left\{4,...,73\right\}.$$

The resulting integer weights can be found in Table 6.

**Table 6:** Integer weights $\mu_{i},\;1\leq i\leq 73$, for the production rules of the grammar ${Ĝ}_{\text{sto}}^{*}$

Nonterminal *Nt*	Integer weights of Rules with Premise *Nt*	
S'	*μ*_1 _: = 10000,	*μ*_2 _: = 212,
	*μ*_3 _: = 3,	
E	*μ*_4 _: = 9788,	*μ*_5 _: = 134,
	*μ*_6 _: = 944,	*μ*_7 _: = 13,
S	*μ*_8 _: = 8559,	*μ*_9 _: = 1304,
	*μ*_10 _: = 126,	*μ*_11 _: = 181,
	*μ*_12 _: = 2,	
C	*μ*_13 _: = 9036,	*μ*_14 _: = 871,
A	*μ*_15 _: = 76300000,	*μ*_16 _: = 4020000,
	*μ*_17 _: = 1860000,	*μ*_18 _: = 3670000,
	*μ*_19 _: = 720000,	*μ*_20 _: = 8580000,
	*μ*_21 _: = 4840000,	
G	*μ*_22 _: = 3038,	*μ*_23 _: = 1884,
	*μ*_24 _: = 3081,	*μ*_25 _: = 1996,
D	*μ*_26 _: = 10000,	*μ*_27 _: = 3896,
B	*μ*_28 _: = 6104,	*μ*_29 _: = 2378,
F	*μ*_30 _: = 5750000,	*μ*_31 _: = 340900000000,
	*μ*_32 _: = 6016000000000000,	*μ*_33 _: = 1211000000000000,
H	*μ*_34 _: = 7987,	*μ*_35 _: = 1608,
P	*μ*_36 _: = 10850000,	*μ*_37 _: = 214400000000,
	*μ*_38 _: = 201100000000,	*μ*_39 _: = 4760000000000000,
Q	*μ*_40 _: = 1713000000000000,	*μ*_41 _: = 828700000000,
R	*μ*_42 _: = 415000000000,	*μ*_43 _: = 585000000000,
V	*μ*_44 _: = 10000,	*μ*_45 _: = 3243,
W	*μ*_46 _: = 10000,	*μ*_47 _: = 3243,
O	*μ*_48 _: = 10000,	*μ*_49 _: = 2928,
J	*μ*_50 _: = 6757,	*μ*_51 _: = 2191,
K	*μ*_52 _: = 7072,	*μ*_53 _: = 2071,
M	*μ*_54 _: = 10000,	*μ*_55 _: = 510,
	*μ*_56 _: = 36,	*μ*_57 _: = 712,
	*μ*_58 _: = 36,	*μ*_59 _: = 3,
X	*μ*_60 _: = 9288,	*μ*_61 _: = 1968,
Z	*μ*_62 _: = 4211,	*μ*_63 _: = 5279,
	*μ*_64 _: = 1119,	*μ*_65 _: = 215,
	*μ*_66 _: = 15,	*μ*_67 _: = 300,
	*μ*_68 _: = 376,	*μ*_69 _: = 80,
	*μ*_70 _: = 15,	*μ*_71 _: = 1,
U	*μ*_72 _: = 7881,	*μ*_73 _: = 1670.

Note that for $i\in \{74,...,105\},\;\mu_{i}:=1$ holds.

#### Transforming Reweighted Grammar into Admissible Specification

Given the reweighted grammar ${Ĝ}_{\text{sto}}^{*}$, we immediately obtain the following admissible specification of the corresponding combinatorial classes (note that this specification has already been simplified by removing classes that are only duplicates of others):

$$\begin{array}{ccc} E_{1}=S\times C, & E_{2}=A\times C,\\ E_{3}=S\times \alpha |, & E_{4}=A\times \alpha |,\\ S_{1}=E\times A, & S_{2}=C\times A, & S_{3}=\alpha |\times A,\\ S_{4}=S\times A, & S_{5}=A\times A,\\ C_{1}=C\times \alpha |, & C_{2}=\alpha |\times \alpha |,\\ A_{1}=\alpha \left(\times A\times \alpha \right), & A_{2}=\alpha \left(\times M\times \alpha \right), & A_{3}=\alpha \left(\times P\times \alpha \right),\\ A_{4}=\alpha \left(\times Q\times \alpha \right), & A_{5}=\alpha \left(\times R\times \alpha \right), & A_{6}=\alpha \left(\times F\times \alpha \right),\\ A_{7}=\alpha \left(\times G\times \alpha \right),\\ G_{1}=A\times \alpha |, & G_{2}=A\times D,\\ G_{3}=\alpha |\times A, & G_{4}=D\times A,\\ D_{1}=B\times \alpha |, & D_{2}=\alpha |\times \alpha |,\\ B_{1}=B\times \alpha |, & B_{2}=\alpha |\times \alpha |,\\ F_{1}=\alpha |\times \alpha |\times \alpha |, & F_{2}=\alpha |\times \alpha |\times \alpha |\times \alpha |, & F_{3}=\alpha |\times \alpha |\times \alpha |\times \alpha |\times H,\\ F_{4}=\alpha |\times \alpha |\times \alpha |\times \alpha |\times \alpha |,\\ H_{1}=H\times \alpha |, & H_{2}=\alpha |\times \alpha |,\\ P_{1}=\alpha |\times A\times \alpha |, & P_{2}=\alpha |\times A\times \alpha |\times \alpha |, & P_{3}=\alpha |\times \alpha |\times A\times \alpha |,\\ P_{4}=\alpha |\times \alpha |\times A\times \alpha |\times \alpha |,\\ Q_{1}=\alpha |\times \alpha |\times O\times \alpha |\times \alpha |, & Q_{2}=\alpha |\times \alpha |\times V\times \alpha |,\\ R_{1}=\alpha |\times O\times \alpha |\times \alpha |, & R_{2}=\alpha |\times \alpha |\times W\times \alpha |,\\ V_{1}=J\times O, & V_{2}=\alpha |\times O,\\ W_{1}=J\times A, & W_{2}=\alpha |\times A,\\ O_{1}=A\times K, & O_{2}=A\times \alpha |,\\ J_{1}=J\times \alpha |, & J_{2}=\alpha |\times \alpha |,\\ K_{1}=K\times \alpha |, & K_{2}=\alpha |\times \alpha |,\\ M_{1}=X\times Z, & M_{2}=X\times X, & M_{3}=X\times A,\\ M_{4}=A\times Z, & M_{5}=A\times X, & M_{6}=A\times A,\\ X_{1}=U\times A, & X_{2}=\alpha |\times A,\\ Z_{1}=X\times Z, & Z_{2}=X\times U, & Z_{3}=X\times \alpha |,\\ Z_{4}=X\times X, & Z_{5}=X\times A,\\ Z_{6}=A\times Z, & Z_{7}=A\times U, & Z_{8}=A\times \alpha |,\\ Z_{9}=A\times X, & Z_{10}=A\times A,\\ U_{1}=U\times \alpha |, & U_{2}=\alpha |\times \alpha |, \end{array}$$

$$\begin{array}{c} S^{\prime }=\mu_{1}\cdot E+\mu_{2}\cdot S+\mu_{3}\cdot A,\\ E=\mu_{4}\cdot E_{1}+\mu_{5}\cdot E_{2}+\mu_{6}\cdot E_{3}+\mu_{7}\cdot E_{4},\\ S=\mu_{8}\cdot S_{1}+\mu_{9}\cdot S_{2}+\mu_{10}\cdot S_{3}+\mu_{11}\cdot S_{4}+\mu_{12}\cdot S_{5},\\ C=\mu_{13}\cdot C_{1}+\mu_{14}\cdot C_{2},\\ A=\mu_{15}\cdot A_{1}+\mu_{16}\cdot A_{2}+\mu_{17}\cdot A_{3}+\mu_{18}\cdot A_{4}+\mu_{19}\cdot A_{5}+\mu_{20}\cdot A_{6}+\mu_{21}\cdot A_{7},\\ G=\mu_{22}\cdot G_{1}+\mu_{23}\cdot G_{2}+\mu_{24}\cdot G_{3}+\mu_{25}\cdot G_{4},\\ D=\mu_{26}\cdot D_{1}+\mu_{27}\cdot D_{2},\\ B=\mu_{28}\cdot B_{1}+\mu_{29}\cdot B_{2},\\ F=\mu_{30}\cdot F_{1}+\mu_{31}\cdot F_{2}+\mu_{32}\cdot F_{3}+\mu_{33}\cdot F_{4},\\ H=\mu_{34}\cdot H_{1}+\mu_{35}\cdot H_{2},\\ P=\mu_{36}\cdot P_{1}+\mu_{37}\cdot P_{2}+\mu_{38}\cdot P_{3}+\mu_{39}\cdot P_{4},\\ Q=\mu_{40}\cdot Q_{1}+\mu_{41}\cdot Q_{2},\\ R=\mu_{42}\cdot R_{1}+\mu_{43}\cdot R_{2},\\ V=\mu_{44}\cdot V_{1}+\mu_{45}\cdot V_{2},\\ W=\mu_{46}\cdot W_{1}+\mu_{47}\cdot W_{2},\\ O=\mu_{48}\cdot O_{1}+\mu_{49}\cdot O_{2},\\ J=\mu_{50}\cdot J_{1}+\mu_{51}\cdot J_{2},\\ K=\mu_{52}\cdot K_{1}+\mu_{53}\cdot K_{2},\\ M=\mu_{54}\cdot M_{1}+\mu_{55}\cdot M_{2}+\mu_{56}\cdot M_{3}+\mu_{57}\cdot M_{4}+\mu_{58}\cdot M_{5}+\mu_{59}\cdot M_{6},\\ X=\mu_{60}\cdot X_{1}+\mu_{61}\cdot X_{2},\\ Z=\mu_{62}\cdot Z_{1}+\mu_{63}\cdot Z_{2}+\mu_{64}\cdot Z_{3}+\mu_{65}\cdot Z_{4}+\mu_{66}\cdot Z_{5}+\mu_{67}\cdot Z_{6}+\mu_{68}\cdot Z_{7}+\mu_{69}\cdot Z_{8}+\mu_{70}\cdot Z_{9}+\mu_{71}\cdot Z_{10},\\ U=\mu_{72}\cdot U_{1}+\mu_{73}\cdot U_{2}. \end{array}$$

Now, this(simplified) specification can easily be transformed into the following recursive form for the function size :

$$\text{size}\left(I,n\right):=\left\{\begin{array}{cc} \mu_{1}\cdot \text{size}\left(E,n\right)+\mu_{2}\cdot \text{size}\left(S,n\right)+\mu_{3}\cdot \text{size}\left(A,n\right) & I=S^{\prime },\\ \text{siz}{\text{e}}_{E}\left(I,n\right) & I\in \left\{E_{i}|1\leq i\leq 4\right\}\;\text{or}\;I=E,\\ \text{siz}{\text{e}}_{S}\left(I,n\right) & I\in \left\{S_{i}|1\leq i\leq 5\right\}\;\text{or}\;I=S,\\ \text{siz}{\text{e}}_{C}\left(I,n\right) & I\in \left\{C_{i}|1\leq i\leq 2\right\}\;\text{or}\;I=C,\\ \text{siz}{\text{e}}_{A}\left(I,n\right) & I\in \left\{A_{i}|1\leq i\leq 7\right\}\;\text{or}\;I=A,\\ \text{siz}{\text{e}}_{G}\left(I,n\right) & I\in \left\{G_{i}|1\leq i\leq 4\right\}\;\text{or}\;I=G,\\ \text{siz}{\text{e}}_{D}\left(I,n\right) & I\in \left\{D_{i}|1\leq i\leq 2\right\}\;\text{or}\;I=D,\\ \text{siz}{\text{e}}_{B}\left(I,n\right) & I\in \left\{B_{i}|1\leq i\leq 2\right\}\;\text{or}\;I=B,\\ \text{siz}{\text{e}}_{F}\left(I,n\right) & I\in \left\{F_{i}|1\leq i\leq 4\right\}\;\text{or}\;I=F,\\ \text{siz}{\text{e}}_{H}\left(I,n\right) & I\in \left\{H_{i}|1\leq i\leq 2\right\}\;\text{or}\;I=H,\\ \text{siz}{\text{e}}_{P}\left(I,n\right) & I\in \left\{P_{i}|1\leq i\leq 4\right\}\;\text{or}\;I=P,\\ \text{siz}{\text{e}}_{Q}\left(I,n\right) & I\in \left\{Q_{i}|1\leq i\leq 2\right\}\;\text{or}\;I=Q,\\ \text{siz}{\text{e}}_{R}\left(I,n\right) & I\in \left\{R_{i}|1\leq i\leq 2\right\}\;\text{or}\;I=R,\\ \text{siz}{\text{e}}_{V}\left(I,n\right) & I\in \left\{V_{i}|1\leq i\leq 2\right\}\;\text{or}\;I=V,\\ \text{siz}{\text{e}}_{W}\left(I,n\right) & I\in \left\{W_{i}|1\leq i\leq 2\right\}\;\text{or}\;I=W,\\ \text{siz}{\text{e}}_{O}\left(I,n\right) & I\in \left\{O_{i}|1\leq i\leq 2\right\}\;\text{or}\;I=O,\\ \text{siz}{\text{e}}_{J}\left(I,n\right) & I\in \left\{J_{i}|1\leq i\leq 2\right\}\;\text{or}\;I=J,\\ \text{siz}{\text{e}}_{K}\left(I,n\right) & I\in \left\{K_{i}|1\leq i\leq 2\right\}\;\text{or}\;I=K,\\ \text{siz}{\text{e}}_{M}\left(I,n\right) & I\in \left\{M_{i}|1\leq i\leq 6\right\}\;\text{or}\;I=M,\\ \text{siz}{\text{e}}_{X}\left(I,n\right) & I\in \left\{X_{i}|1\leq i\leq 2\right\}\;\text{or}\;I=X,\\ \text{siz}{\text{e}}_{Z}\left(I,n\right) & I\in \left\{Z_{i}|1\leq i\leq 10\right\}\;\text{or}\;I=Z,\\ \text{siz}{\text{e}}_{U}\left(I,n\right) & I\in \left\{U_{i}|1\leq i\leq 2\right\}\;\text{or}\;I=U,\\ 0 & \text{else}, \end{array}\right)$$

where

sizeE(I,n):=∑j=1n-1 size(S,j)⋅ size(C,n-j)I=E1,∑j=1n-1 size(A,j)⋅ size(C,n-j)I=E2,size(S,n-1)I=E3,size(A,n-1)I=E4,μ4⋅ size(E1,n)+μ5⋅ size(E2,n)+μ6⋅ size(E3,n)+μ7⋅ size(E4,n)I=E,0else,

sizeS(I,n):=∑j=1n-1 size(E,j)⋅ size(A,n-j)I=S1,∑j=1n-1 size(C,j)⋅ size(A,n-j)I=S2,size(A,n-1)I=S3,∑j=1n-1 size(S,j)⋅ size(A,n-j)I=S4,∑j=1n-1 size(A,j)⋅ size(A,n-j)I=S5,μ8⋅ size(S1,n)+μ9⋅ size(S2,n)+μ10⋅ size(S3,n)+μ11⋅ size(S4,n)+μ12⋅ size(S5,n)I=S,0else,

$$\text{siz}{\text{e}}_{C}\left(I,n\right):=\left\{\begin{array}{cc} \text{size}\left(C,n-1\right) & I=C_{1},\\ 1 & I=C_{2},\;\text{and}\;n=2,\\ \mu_{13}\cdot \text{size}\left(C_{1},n\right)+\mu_{14}\cdot \text{size}\left(C_{2},n\right) & I=C,\\ 0 & \text{else}, \end{array}\right)$$

$${\mathrm{size}}_{A}\left(I,n\right):=\left\{\begin{array}{cc} \mathrm{size}\left(A,n-2\right)\; & I=A_{1},\\ \mathrm{size}\left(M,n-2\right)\; & I=A_{2},\\ \mathrm{size}\left(P,n-2\right)\; & I=A_{3},\\ \mathrm{size}\left(Q,n-2\right)\; & I=A_{4},\\ \mathrm{size}\left(R,n-2\right)\; & I=A_{5},\\ \mathrm{size}\left(F,n-2\right)\; & I=A_{6},\\ \mathrm{size}\left(G,n-2\right)\; & I=A_{7},\\ \mu_{15}\cdot \mathrm{size}\left(A_{1},n\right)+\mu_{16}\cdot \mathrm{size}\left(A_{2},n\right)+\mu_{17}\cdot \mathrm{size}\left(A_{3},n\right)+\mu_{18}\cdot \mathrm{size}\left(A_{4},n\right)\;\\ +\mu_{19}\cdot \mathrm{size}\left(A_{5},n\right)+\mu_{20}\cdot \mathrm{size}\left(A_{6},n\right)+\mu_{21}\cdot \mathrm{size}\left(A_{7},n\right)\; & I=A,\\ 0\; & \text{else},\\ \; \end{array}\right)$$

sizeG(I,n):=size(A,n-1)I=G1,∑j=1n-1 size(A,j)⋅ size(D,n-j)I=G2,size(A,n-1)I=G3,∑j=1n-1 size(D,j)⋅ size(A,n-j)I=G4,μ22⋅ size(G1,n)+μ23⋅ size(G2,n)+μ24⋅ size(G3,n)+μ25⋅ size(G4,n)I=G,0else,

$$\text{siz}{\text{e}}_{D}\left(I,n\right):=\left\{\begin{array}{cc} \text{size}\left(B,n-1\right) & I=D_{1},\\ 1 & I=D_{2}\;\text{and}\;n=2,\\ \mu_{26}\cdot \text{size}\left(D_{1,n}\right)+\mu_{27}\cdot \text{size}\left(D_{2,n}\right) & I=D,\\ 0 & \text{else}, \end{array}\right)$$

$$\text{siz}{\text{e}}_{B}\left(I,n\right):=\left\{\begin{array}{cc} \text{size}\left(B,n-1\right) & I=B_{1},\\ 1 & I=B_{2}\;\text{and}\;n=2,\\ \mu_{28}\cdot \text{size}\left(B_{1,n}\right)+\mu_{29}\cdot \text{size}\left(B_{2,n}\right) & I=B,\\ 0 & \text{else}, \end{array}\right)$$

$$\text{siz}{\text{e}}_{F}\left(I,n\right):=\left\{\begin{array}{cc} 1 & I=F_{1}\;\text{and}\;n=3,\\ 1 & I=F_{2}\;\text{and}\;n=4,\\ \text{size}\left(H,n-4\right) & I=F_{3},\\ 1 & I=F_{4}\;\text{and}\;n=5,\\ \mu_{30}\cdot \text{size}\left(F_{1},n\right)+\mu_{31}\cdot \text{size}\left(F_{2},n\right) & \\ +\mu_{32}\cdot \text{size}\left(F_{3},n\right)+\mu_{33}\cdot \text{size}\left(F_{4},n\right) & I=F,\\ 0 & \text{else}, \end{array}\right)$$

$$\text{siz}{\text{e}}_{H}\left(I,n\right):=\left\{\begin{array}{cc} \text{size}\left(H,n-1\right) & I=H_{1},\\ 1 & I=H_{2},\;\text{and}\;n=2\\ \mu_{34}\cdot \text{size}\left(H_{1},n\right)+\mu_{35}\cdot \text{size}\left(H_{2},n\right) & I=H,\\ 0 & \text{else}, \end{array}\right)$$

$$\text{siz}{\text{e}}_{P}\left(I,n\right):=\left\{\begin{array}{cc} \text{size}\left(A,n-2\right) & I=P_{1},\\ \text{size}\left(A,n-3\right) & I=P_{2},\\ \text{size}\left(A,n-3\right) & I=P_{3},\\ \text{size}\left(A,n-4\right) & I=P_{4},\\ \mu_{36}\cdot \text{size}\left(P_{1},n\right)+\mu_{37}\cdot \text{size}\left(P_{2},n\right)+\mu_{38}\cdot \text{size}\left(P_{3},n\right)+\mu_{39}\cdot \text{size}\left(P_{4},n\right) & I=P,\\ 0 & \text{else}, \end{array}\right)$$

$$\text{siz}{\text{e}}_{Q}\left(I,n\right):=\left\{\begin{array}{cc} \text{size}\left(O,n-4\right) & I=Q_{1},\\ \text{size}\left(V,n-3\right) & I=Q_{2},\\ \mu_{40}\cdot \text{size}\left(Q_{1,n}\right)+\mu_{41}\cdot \text{size}\left(Q_{2,n}\right) & I=Q,\\ 0 & \text{else}, \end{array}\right)$$

$$\text{siz}{\text{e}}_{R}\left(I,n\right):=\left\{\begin{array}{cc} \text{size}\left(O,n-3\right) & I=R_{1},\\ \text{size}\left(W,n-3\right) & I=R_{2},\\ \mu_{42}\cdot \text{size}\left(R_{1,n}\right)+\mu_{43}\cdot \text{size}\left(R_{2,n}\right) & I=R,\\ 0 & \text{else}, \end{array}\right)$$

$$\text{siz}{\text{e}}_{V}\left(I,n\right):=\left\{\begin{array}{cc} \sum_{j=1}^{n-1}\text{size}\left(J,j\right)\cdot \text{size}\left(O,n-j\right) & I=V\\ \text{size}\left(O,n-1\right) & I=V_{2},\\ \mu_{44}\cdot \text{size}\left(V_{1},n\right)+\mu_{45}\cdot \text{size}\left(V_{2},n\right) & I=V,\\ 0 & \text{else}, \end{array}\right)$$

$$\text{siz}{\text{e}}_{W}\left(I,n\right):=\left\{\begin{array}{cc} \sum_{j=1}^{n-1}\text{size}\left(J,j\right)\cdot \text{size}\left(A,n-j\right) & I=W_{1}\\ \text{size}\left(A,n-1\right) & I=W_{2},\\ \mu_{46}\cdot \text{size}\left(W_{1},n\right)+\mu_{47}\cdot \text{size}\left(W_{2},n\right) & I=W,\\ 0 & \text{else}, \end{array}\right)$$

$$\text{siz}{\text{e}}_{O}\left(I,n\right):=\left\{\begin{array}{cc} \sum_{j=1}^{n-1}\text{size}\left(A,j\right)\cdot \text{size}\left(K,n-j\right) & I=O_{1}\\ \text{size}\left(A,n-1\right) & I=O_{2},\\ \mu_{48}\cdot \text{size}\left(O_{1},n\right)+\mu_{49}\cdot \text{size}\left(O_{2},n\right) & I=O,\\ 0 & \text{else}, \end{array}\right)$$

$$\text{siz}{\text{e}}_{J}\left(I,n\right):=\left\{\begin{array}{cc} \text{size}\left(J,n-1\right) & I=J_{1},\\ 1 & I=J_{2}\;\text{and}\;n=2,\\ \mu_{50}\cdot \text{size}\left(J_{1},n\right)+\mu_{51}\cdot \text{size}\left(J_{2},n\right) & I=J\\ 0 & \text{else}, \end{array}\right)$$

$$\text{siz}{\text{e}}_{K}\left(I,n\right):=\left\{\begin{array}{cc} \text{size}\left(K,n-1\right) & I=K_{1},\\ 1 & I=J_{2}\;\text{and}\;n=2,\\ \mu_{52}\cdot \text{size}\left(K_{1},n\right)+\mu_{53}\cdot \text{size}\left(K_{2},n\right) & I=K,\\ 0 & \text{else}, \end{array}\right)$$

sizeM(I,n):=∑j=1n-1 size(X,j)⋅ size(Z,n-j)I=M1,∑j=1n-1 size(X,j)⋅ size(X,n-j)I=M2,∑j=1n-1 size(X,j)⋅ size(A,n-j)I=M3,∑j=1n-1 size(A,j)⋅ size(Z,n-j)I=M4,∑j=1n-1 size(A,j)⋅ size(X,n-j)I=M5,∑j=1n-1 size(A,j)⋅ size(A,n-j)I=M6,μ54⋅ size(M1,n)+μ55⋅ size(M2,n)+μ56⋅ size(M3,n)+μ57⋅ size(M4,n)+μ58⋅ size(M5,n)+μ59⋅ size(M6,n)I=M,0else,

$$\text{siz}{\text{e}}_{X}\left(I,n\right):=\left\{\begin{array}{cc} \sum_{j=1}^{n-1}\text{size}\left(U,j\right)\cdot \text{size}\left(A,n-j\right) & I=X_{1}\\ \text{size}\left(A,n-1\right) & I=X_{2},\\ \mu_{60}\cdot \text{size}\left(X_{1},n\right)+\mu_{61}\cdot \text{size}\left(X_{2},n\right) & I=X,\\ 0 & \text{else}, \end{array}\right)$$

$$\text{siz}{\text{e}}_{z}\left(I,n\right):=\left\{\begin{array}{cc} \sum_{j=1}^{n-1}\text{size}\left(X,j\right)\cdot \text{size}\left(Z,n-j\right) & I=Z_{1},\\ \sum_{j=1}^{n-1}\text{size}\left(X,j\right)\cdot \text{size}\left(U,n-j\right) & I=Z_{2},\\ \text{size}\left(X,n-1\right) & I=Z_{3},\\ \sum_{j=1}^{n-1}\text{size}\left(X,j\right)\cdot \text{size}\left(X,n-j\right) & I=Z_{4},\\ \sum_{j=1}^{n-1}\text{size}\left(X,j\right)\cdot \text{size}\left(A,n-j\right) & I=Z_{5},\\ \sum_{j=1}^{n-1}\text{size}\left(A,j\right)\cdot \text{size}\left(Z,n-j\right) & I=Z_{6},\\ \sum_{j=1}^{n-1}\text{size}\left(A,j\right)\cdot \text{size}\left(U,n-j\right) & I=Z_{7},\\ \text{size}\left(A,n-1\right) & I=Z_{8},\\ \sum_{j=1}^{n-1}\text{size}\left(A,j\right)\cdot \text{size}\left(A,n-j\right) & I=Z_{9},\\ \sum_{j=1}^{n-1}\text{size}\left(A,j\right)\cdot \text{size}\left(A,n-j\right) & I=Z_{10},\\ \mu_{62}\cdot \text{size}\left(Z_{1},n\right)+\mu_{63}\cdot \text{size}\left(Z_{2},n\right)+\mu_{64}\cdot \text{size}\left(Z_{3},n\right)+\mu_{65}\cdot \text{size}\left(Z_{4},n\right) & \\ +\mu_{66}\cdot \text{size}\left(Z_{5},n\right)+\mu_{67}\cdot \text{size}\left(Z_{6},n\right)+\mu_{68}\cdot \text{size}\left(Z_{7},n\right)+\mu_{69}\cdot \text{size}\left(Z_{8},n\right) & \\ +\mu_{70}\cdot \text{size}\left(Z_{9},n\right)+\mu_{71}\cdot \text{size}\left(Z_{10},n\right) & I=Z,\\ 0 & \text{else}, \end{array}\right)$$

$$\text{siz}{\text{e}}_{U}\left(I,n\right):=\left\{\begin{array}{cc} \text{size}\left(U,n-1\right) & I=U_{1},\\ \text{1} & I=U_{2},\;\text{and}\;n=\text{2}\\ \mu_{72}\cdot \text{size}\left(U_{1},n\right)+\mu_{73}\cdot \text{size}\left(U_{2},n\right) & I=U,\\ 0 & \text{else}. \end{array}\right)$$

From those recurrences, the desired algorithm can easily be constructed. As the complete presentation of this algorithm would be too comprehensive, we decided to omit it and instead refer to Algorithms 1 to 4 and 6 given in [20], since for the construction of our unranking algorithm, we had to use exactly these Algorithms as subroutines.
